# The Impact of Electronic Health Records on Nurses and Nursing Care in Low‐ and Middle‐Income Countries: A Scoping Review

**DOI:** 10.1002/nop2.70649

**Published:** 2026-06-25

**Authors:** Wahab Osman, Stephanie Asah‐Ofori, Alexandra Harris, Vida N. Yakong, Kimberley Widger, Charlene H. Chu

**Affiliations:** ^1^ Lawrence Bloomberg Faculty of Nursing University of Toronto Toronto Ontario Canada; ^2^ School of Nursing and Midwifery University for Development Studies Tamale Ghana; ^3^ Ghana College of Nurses and Midwives (GCNM) Accra Ghana; ^4^ Unity Health Toronto Toronto Ontario Canada; ^5^ Li Ka Shing Knowledge Institute St. Michael's Hospital Toronto Ontario Canada; ^6^ Hospital for Sick Children Toronto Ontario Canada; ^7^ Institute for Clinical and Evaluative Science Toronto Ontario Canada; ^8^ KITE Research Institute, Toronto Rehabilitation Institute University Health Network Toronto Ontario Canada; ^9^ Institute for Life Course and Aging University of Toronto Toronto Ontario Canada

**Keywords:** digital health, eHealth, electronic health records, health informatics, low‐income countries, middle‐income countries, nurses, nursing, nursing documentation, nursing informatics

## Abstract

**Aim:**

To map evidence and identify research gaps from primary studies on the impact of electronic health records (EHRs) on nurses and nursing care in low‐ and middle‐income countries (LMICs) from 2015 to 2024.

**Design:**

A scoping literature review.

**Review Methods:**

Guided by the Joanna Briggs Institute (JBI) guidelines for evidence synthesis, two reviewers conducted screening and data extraction using Covidence.

**Data Sources:**

A systematic search of Medline, Embase, CINAHL, Scopus, Web of Science, and WHO Global Index Medicus databases was conducted in May 2024.

**Results:**

Of 6122 studies, 41 were included. Most employed quantitative methods, but often did not use validated tools or theoretical frameworks. EHRs improved documentation, but some quality deficits remain. Depersonalized care, workflow disruptions, reduced nurse–patient interaction, and technostress were also reported. Regional research output disparities emerged.

**Conclusions:**

The evidence demonstrates that EHRs enhanced nursing documentation and data access, yet were also associated with data quality deficits, increased burden, workflow disruption, technostress, and depersonalized care in LMICs. The LMIC regional research disparities and theoretically underdeveloped evidence base, particularly from Sub‐Saharan Africa, necessitate nurse‐led investigations of documentation quality, workload, care disruption and nurse well‐being to inform context‐sensitive practice, education and policy.

**Implication for Nursing and/or Patient Care:**

The findings will guide nurses and policymakers in this region to optimize the use of digital health systems, promote equitable access, stimulate further research, provide solutions, and support effective ehealth transformation in LMICs.

**Impact:**

This review identifies critical gaps in current research on electronic health records, highlighting both benefits and drawbacks for nurses and nursing care. The findings will guide nurses and policymakers in this region to improve digital systems implementation and inform future nursing research and policy development.

**Reporting Method:**

Reported in accordance with the Preferred Reporting Items for Systematic Reviews and Meta‐Analyses extension for Scoping Reviews (PRISMA‐ScR).

**Patient or Public Contribution:**

No Patient or Public Contribution.

**Review Protocol Registration:**

DOI: https://doi.org/10.17605/osf.io/gntks

## 1 Introduction

The integration of electronic health records (EHRs) into healthcare systems globally represents a pivotal transition from traditional paper‐based recording of patient care to digital platforms. This shift brings with it a range of potential benefits and challenges for healthcare delivery (International Standards Organization [ISO] 2024; World Health Organization [WHO] 2019). EHRs have been shown to enhance documentation practices, improve clinical decision‐making, and streamline communication between nurses, healthcare team members and patients (Hants et al. 2023). This transformation is especially important in the context of nursing, where studies have estimated that nurses spend between 25% and 55% of their shift time in care documentation (Khan et al. 2022; Yen et al. 2018).

The International Standards Organization (ISO) describes EHRs as a comprehensive digital repository of a patient's medical information that documents their entire healthcare journey in real time (ISO 2024). EHR systems contain data on patients' medical histories, diagnoses, medications, treatment plans, immunizations, allergies, radiology images, laboratory test results, treatment, and clinical care notes of health professionals (Office of the National Coordinator for Health Information Technology [ONC HIT] 2024). The systems not only store patient data but may also incorporate order entry systems for prescribing tests, medications and treatments; decision support systems providing evidence‐based care recommendations; and embedded communication tools facilitating coordination among healthcare practitioners (ISO 2024). By improving these processes, EHRs enhance the quality of care delivered by healthcare professionals, including nurses.

Nurses are the largest group of healthcare professionals globally and hence are the primary generators and users of patient care data within EHR systems (Fraczkowski et al. 2020). EHRs offer numerous benefits to nursing, including improved accuracy and completeness of patient data, enhanced communication and collaboration among healthcare providers, and increased efficiency in nursing workflows (Pérez‐Martí et al. 2022; Shala et al. 2021). The impact of EHR utilization on nursing care practices is multifaceted in the literature; it influences documentation practices (Reiner 2021), clinical decision‐making (Wu et al. 2020), nurse–patient communication (Forde‐Johnston et al. 2023) and workload (Naamneh and Bodas 2024).

EHRs have demonstrated significant potential to enhance nursing efficiency and patient care quality. They have been shown to improve the accuracy and efficiency of documentation, reducing the likelihood of errors and enhancing the quality of patient records (Misto et al. 2019; Shiells et al. 2019). EHRs can reduce documentation time between 18% ‐ 60% (Venkateswaran et al. 2022), allowing more time for direct patient care (Khan et al. 2022). EHRs also facilitate adherence to clinical guidelines, contributing to improved patient outcomes (Pearkao et al. 2023). They can enhance healthcare access and nursing efficiency through timely information access, clinical decision support, and improved communication among providers (Venkateswaran et al. 2022; Ye et al. 2023). By streamlining processes, EHRs can potentially reduce burnout and enhance job satisfaction among nurses (McBride et al. 2023). Additionally, EHRs support data collection for public health surveillance, policymaking, and research (Schenk et al. 2018). However, some studies report increased documentation time and potential disruptions to nurse–patient communication as unintended consequences of EHR use (Lee 2021; Schenk et al. 2018).

Previous literature reviews on nurses use of EHRs point out the presence of significant knowledge gap concerning the impact of EHRs on nursing practice, with their findings highlighting the fact that previous research have been predominantly conducted in high‐income countries and focused on implementation and adoption challenges (Forde‐Johnston et al. 2023; Jedwab et al. 2023; McBride et al. 2023). Similarly in LMICs, literature have mainly addressed EHR user satisfaction, pre‐adoption perceptions and implementation barriers, often involving a mix of healthcare professionals, with limited focus on nurses and nursing (Adedeji et al. 2023; Kumar and Mostafa 2020; Zharima et al. 2023). This indicates a notable gap in health literature regarding research and evidence from LMIC contexts on this topic.

In the resource‐limited settings of low‐ and middle‐income countries (LMICs) (World Bank Group 2024a), EHR implementation faces challenges such as limited technology access, insufficient training and inadequate resources (Adedeji et al. 2023; Rayan 2020). Nurses' perspective on the impact of EHR use on their work, following its integration, remains underexplored in these settings (Kumar and Mostafa 2020; Ndung'u and Signé 2020).

To address the identified knowledge and research gap and contribute to the broader understanding of how EHRs impact nurses and nursing practice in the low‐resource settings of LMICs, a comprehensive synthesis of existing evidence using a scoping literature review methodology is warranted. There is therefore the need to answer the question of ‘what is known regarding the impact of EHR use on nurses and nursing care in LMICs’ based on evidence from primary research.

As health systems in these resource‐limited settings undergo rapid digitization, understanding the impact of EHRs on nurses and nursing practice provides evidence for system optimization, policy refinement, implementation success and research. It also ensures equitable access to the benefits of digital health transformation and the drive toward the achievement of the UN Sustainable Development Goal 3 (SDG 3) United Nations Sustainable Development Goal 3 (SDG 3).

## 2 Review Aim and Questions

This scoping literature review therefore aimed to map evidence from primary studies on the impact of EHRs on nurses and nursing care in LMICs from 2015 to 2024. Secondarily, it sought to identify knowledge and research gaps as well as the sub‐regional distribution of primary studies on EHRs and nursing care.

Specifically, the review sought to answer the following questions:

1. In which LMIC regions and practice settings has the impact of EHRs on nurses and nursing care been studied?
2. What study designs, data collection methods, and analytical approaches have been used to investigate EHR impacts on nurses and nursing care in LMICs?
3. Which domains of nursing practice and nursing‐sensitive outcomes have been examined in relation to EHR use?
4. To what extent do studies in this area apply validated instruments and theoretical or conceptual frameworks to examine EHR impacts on nurses and nursing care?

## 3 Review Methods

### 3.1 Design

This scoping literature review followed the Joanna Briggs Institute Manual for Evidence Synthesis for scoping reviews (Peters et al. 2020; Pollock et al. 2023). A scoping review methodology was used because of the inadequate evidence on the topic and the quest to identify gaps in research on the impact of EHRs on nurses and nursing care in LMICs. Consistent with JBI and PRISMA ScR guidelines for scoping literature reviews (Peters et al. 2022; Tricco et al. 2018), an a priori protocol developed for the first author's doctoral research guided this review. Consistent with JBI and other literature review guidelines for scoping literature reviews (Peters et al. 2022; Tricco et al. 2018), an a priori protocol developed for the first author's doctoral research guided this review. A search of JBI Evidence Synthesis, the Open Science Framework (OSF) registry, and PROSPERO did not identify any registered protocol or ongoing review on this topic. Consequently, our protocol was registered in the OSF registry (DOI: https://doi.org/10.17605/OSF.IO/GNTKS).

### 3.2 Search Methods

A comprehensive literature search was executed in May 2024 and conducted in six electronic databases: Ovid Medline, Ovid Embase, Web of Science, Scopus, the Cumulative Index of Nursing and Allied Health Literature (CINAHL), and the World Health Organization (WHO) Medicus Index. The search strategy (See Appendix S1) was developed in collaboration with an experienced librarian with expertise in nursing and health literature. The strategy was adapted to the requirements of each database. Initial search terms were formulated by identifying relevant keywords and phrases within the Medical Subject Headings (MeSH), including electronic health records and their synonyms; nurses and nursing care; and low‐ and middle‐income countries as classified by the World Bank (World Bank Group 2024a). The process was iterative, incorporating additional keywords and subject headings as needed.

### 3.3 Eligibility Criteria

Primary research papers were included if they reported on the following population, concept, and context (PCC) criteria (Peters et al. 2022):

#### 3.3.1 Population

The included studies had to involve nurses of any cadre as the study population. Mixed samples comprising nurses and other health professionals were eligible only when nurse‐specific data could be disaggregated from those of other participants.

#### 3.3.2 Concept

In this review, the concept of ‘impact on nurses and nursing care’ was defined as the multidimensional consequences of EHR use for both nurses and the delivery of nursing care in LMIC settings. Specifically, we considered impacts on: (1) Nurses' work processes and workload, (2) The quality of nursing documentation and information, (3) Clinical care coordination and interprofessional communication, (4) Nurse–patient interaction and relational aspects of care, (5) Nurses' satisfaction and professional experience with EHRs, and (6) Psychological well‐being and technostress in nurses. Within the last domain, we were attentive to EHR‐related workload, stress and technostress as potential contributors to outcomes such as satisfaction with digital health tools, intention to leave and burnout‐related experiences (McBride et al. 2023). These domains were specified in advance in the protocol to guide study selection and data charting, while remaining open to additional subdomains that emerged inductively from the included primary studies, consistent with JBI guidance for scoping reviews (Peters et al. 2022).

#### 3.3.3 Context

The research context is any LMIC country as categorized by the World Bank (World Bank Group 2024a). Any practice setting in LMICs where nurses used any form of EHRs, including hospitals, immunization centres and long‐term care or aged homes. This review only considered full‐text primary quantitative, qualitative and mixed‐method studies that were peer‐reviewed and published in the English language, between 2015 to 2024. The chosen timeframe is justified by the gradual increase in investment and implementation of EHRs and other health information systems in LMICs over the past decade (African Union Commission 2016; Kumar and Mostafa 2019).

Papers were excluded if they were opinion pieces, commentaries, letters to the editor, book chapters, conference abstracts, protocols, literature reviews, or grey literature. This was consistent with our scoping review protocol's focus on primary studies, aimed to map evidence and identify gaps based on our predefined PCC‐based eligibility criteria (Peters et al. 2022; Tricco et al. 2018). This decision ensured standardization and minimized redundancy while prioritizing peer‐reviewed empirical data relevant to EHR impacts on nursing in LMICs.

Also, papers that were solely conducted in high‐income countries, as categorized by the World Bank (World Bank Group 2024a), were also excluded. We restricted inclusion to English‐language publications because the review team did not have sufficient linguistic coverage or funding for translation services to reliably screen and extract data from non‐English studies. This pragmatic decision is consistent with common practice in evidence syntheses (Peters et al. 2022).

### 3.4 Search Outcome

**FIGURE 1:**
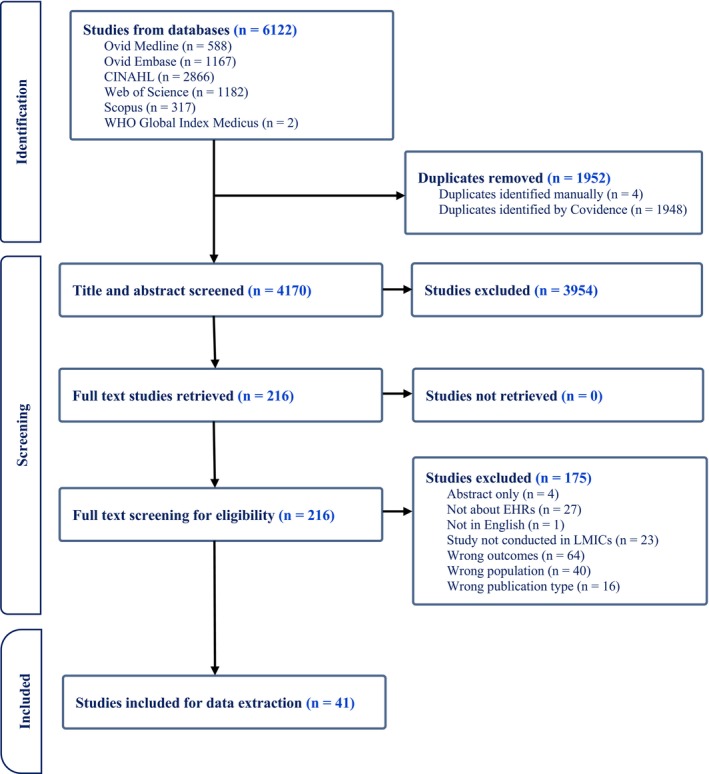
PRISMA Flow Diagram of Literature Screening Process.

The search identified a total of 6122 potential papers from the six databases. The search results were uploaded into Covidence online literature review management platform, where duplicate papers were removed before screening. Title and abstract screening were undertaken by two reviewers working independently. Consensus meetings were held regularly between the reviewers to resolve any disagreements during the screening process. After the screening phase, the full texts of potentially relevant papers were retrieved and assessed independently by the two reviewers for final inclusion based on the predefined inclusion and exclusion criteria. Papers with two‐reviewer agreement were accepted and included. Any disagreements were resolved through reviewer discussions and the involvement of a third reviewer. Consequently, 41 studies were found to have successfully met the inclusion criteria (See Figure 1). Data were thus extracted from the 41 included studies using Covidence and exported into an Excel document for analysis.

### 3.5 Critical Appraisal

Though not mandatory for scoping literature reviews (Peters et al. 2020), the authors undertook quality appraisal of the included papers using the Mixed Method Appraisal Tool version 2018 (MMAT version 2018) by Hong et al. (2018) to understand the methodological quality of the included papers (See Appendix S2). Two reviewers independently assessed each paper, and a consensus was reached upon discussions when there were scoring disagreements. The assessment, however, did not inform the inclusion or exclusion of any study in the final analysis.

### 3.6 Data Extraction

Two reviewers conducted data extraction independently in Covidence using a data extraction template. The template was developed and piloted by the reviewers prior to data extraction. Data from five randomly selected articles were extracted by the two reviewers independently to pilot the template and make the necessary changes to ensure the relevant information was captured. Subsequently, data were extracted from the remaining papers. The data extracted included the author and year of publication, title, country and world region of study, study aim, methods (study setting, population, study design, data collection methods and instrument, and analysis type), name of theory/model used, and findings related to the impact of the use of EHRs on nurses and nursing care. Discussions were then held among the reviewers to resolve disagreements in the extracted data through consensus.

### 3.7 Data Synthesis

A descriptive content narrative approach to data synthesis and analysis was employed. It involved organizing, tabulating, narrating, and discussing findings from the included studies. The PRISMA‐ScR guidelines by Tricco et al. (2018) guided the reporting (See Appendix S3).

## 4 Results

### 4.1 Characteristics of Included Studies

**FIGURE 2:**
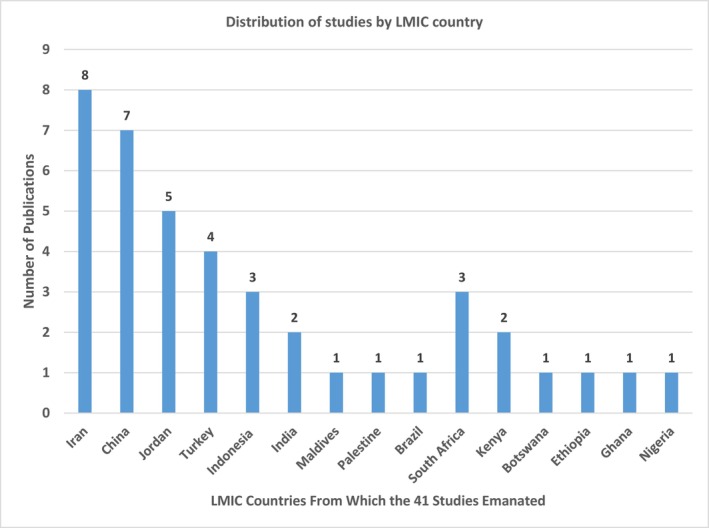
Number of Included Publications per LMIC Country (*n* = 41).

Table 1 summarizes the characteristics of the 41 studies selected for this review. Research activities on the impact of EHRs on nursing increased from 5 studies (12.2%) in 2015 to 10 studies (24.4%) in 2022. Quantitative designs (58.5%) such as cross‐sectional studies were predominant followed by qualitative studies (29.3%), with focus groups as the main design. Most studies (*n* = 27, 63.0%) did not report the use of any theory or model. Most studies (*n* = 32, 78.0%) did not use validated instruments to measure outcomes. Most studies (*n* = 35, 85.4%) were judged to be of high methodological quality, with one (2.4%) being of poor quality (See Appendix S2). The most frequently used theories were the Information Systems Success model (4 studies) by DeLone and McLean (Delone and McLean 2003, 1992), and the Technology Acceptance Model by Davis and colleagues (Davis 1989; Venkatesh and Davis 2000) (Appendix S4). As shown in Figure 2, the included studies were from diverse countries and regions within LMICs, with most conducted in Iran (*n* = 8, 19.5%) and China (*n* = 7, 17.1%). Majority of the studies (18, 43.9%) were from Middle‐Eastern countries, with only 9 (22.0%) from Sub‐Saharan African countries.

**TABLE 1:** Characteristics of included studies (*n* = 41).

Authors and year	Country	Aim	Methods			QA score (MMAT version 2018)
			Study setting and participants	Study design and data collection	Data analysis	
Abed et al. (2022)	Jordan	To determine nurses' attitudes toward using EHRs in their daily work and to address factors that affect their experience in using EHRs during the COVID‐19 pandemic.	Hospital 130 Nurses	Quantitative: Cross‐sectional survey The Nurses Attitudes toward Computerization (NATC) Questionnaire by Stronge and Brodt (1985).	Quantitative: Descriptive and inferential statistics	5
Adereti and Olaogun (2019)	Nigeria	To evaluate the effect of electronic and paper‐based standardized nursing care plans (SNCPs) use on the quality of nurses' documentation	Hospital 406 Nurses	Quantitative: Non‐randomized trial. Pre and posttest questionnaire and checklist: (1) Müller‐Staub et al. (2008) quality of nursing diagnoses, interventions and out‐comes (Q‐DIO) instrument (2) Author‐developed checklist using the Standardized nursing language (SNLs).	Quantitative: descriptive and inferential statistics	3
Akhu‐Zaheya et al. (2018)	Jordan	To assess and compare the quality of nursing documentation of paper‐based versus EHR in terms of content, process and structure.	Hospital 434 Nursing care records (for both paper‐based and EHRs)	Quantitative: Cross sectional retrospective surveys Data collected with the Cat‐Ch‐Ing audit instrument developed by Björvell et al. (2000), based on the VIPS model	Quantitative: Descriptive and inferential statistics	5
Alfuqaha et al. (2022)	Jordan	Explore the perceived level of usefulness and ease of use of electronic health records (EHRs) among nurses and other healthcare providers (HCPs) during the COVID‐19 pandemic in Jordan.	Hospital 300^a^ (150 Nurses)	Quantitative: Cross sectional surveys Data collected using an adapted questionnaire from TAM (Davis 1989).	Quantitative: Descriptive and inferential statistics	5
Arikan et al. (2022)	Turkey	To investigate the barriers to implementation of electronic health record systems from the perspective of nurses.	Hospital 160 Nurses (156 used for analysis)	Quantitative: Cross sectional surveys Data collected using an author developed questionnaire.	Quantitative: Descriptive and inferential statistics	5
Attafuah et al. (2022)	Ghana	To assess the satisfaction of health leaders with the electronic health record (EHR) from the end‐user point of view.	9 Hospitals 100^a^ (65 Nurses)	Qualitative: Focus group discussion (FGD)	Qualitative: Thematic analysis	3
Bei‐lei et al. (2019)	China	To survey the types of Electronic Nursing Records used and to explore nurses' perceptions in the hospitals in Henan Province, China.	16 Hospitals 3586 Nurses	Quantitative: Cross‐sectional surveys. Data collected using an online questionnaire	Quantitative: Descriptive and inferential statistics	4
Cheung and Yip (2024)	China	To evaluate the differences in documentation completeness between paper and electronic tablet‐based resuscitation records.	Hospital 2 Nurses and 528 Nursing care records (176 each for Pre‐App Paper, Post‐App Paper and Post‐App Electronic).	Mixed method: Quantitative—qualitative Data collected using a checklist and interview guide	Qualitative: Content analysis Quantitative: Descriptive and inferential statistics. Triangulation	5
Cohen et al. (2016)	South Africa	To determine which attributes of an integrated hospital information system (HIS) are most important to satisfaction and productivity of nurses who use the system in day‐to‐day clinical practice in a public hospital in South Africa.	Hospital 154 Nurses	Mixed methods: Quantitative—qualitative Data collected using a semi‐structured questionnaire	Quantitative: Descriptive and inferential statistics Qualitative: Thematic analysis	4
Dolan et al. (2023)	Kenya	To describe how healthcare workers in Kenya had integrated an electronic immunization registry into their immunization clinic workflows.	Immunization centres 12 Nurses	Qualitative: Interviews Data collected using an interview guide based on the expanded usability heuristics by Nielsen (1994).	Qualitative: Thematic analysis	5
Farshi et al. (2015)	Iran	To compare electronic and manual nursing record systems from the perspective of nurses working in NICUs in two healthcare centres.	Hospital 63 Nurses	Quantitative: Cross sectional surveys Data collected using an author‐developed questionnaire	Quantitative: Descriptive and inferential statistics	4
Faujdar et al. (2021)	India	To assess the perceptions of various stakeholders about the implementation of the Integrated Health Information System for Primary Health Care (IHIS4PHC)	Outpatient setting 6^a^ Key informants (2 Nurses and midwives)	Qualitative: Ethnography Key informant interviews for Nurses/Midwives	Qualitative: Thematic analysis	5
Firouzeh et al. (2017)	Iran	To compare the quality of nursing documentation in electronic and paper‐based systems.	Hospital 120 Nursing care records	Quantitative: Cross sectional retrospective surveys Data was collected using a checklist	Quantitative: Descriptive and inferential statistics	5
Galani et al. (2021)	Botswana	To investigate barriers to the effective use of electronic health records (EHR) to improve health outcomes for HIV/AIDS care treatment in health centres in rural areas of Botswana	Hospital 45 Nurses	Qualitative: Constructivist grounded theory Data collected using a semi‐structured guide	Qualitative: Thematic analysis	5
Gomes et al. (2019)	Brazil	To analyse the nurse's viewpoint regarding both implementation and use of the Electronic Citizen Record (ECR) in nursing care	Hospital 11 Nurses	Qualitative: Interviews Data collected using semi‐structured interview guide	Qualitative: Thematic analysis	5
Hariyati et al. (2020)	Indonesia	To explore and describe the usability and satisfaction of using electronic documentation.	Hospital Qualitative—8 Nurses Quantitative – 219 Nurses	Mixed method: Quantitative—Qualitative Data collected using: Quantitative: an adapted questionnaire from Abdrbo (2017). Qualitative: Focus groups discussion guide	Quantitative: Descriptive and inferential statistics Qualitative: Content analysis	5
Heidarizadeh et al. (2017)	Iran	To explore nurses' perceptions and challenges in using an electronic nursing documentation system.	Hospital 18 Nurses	Qualitative: Interviews Data collected using a semi‐structured interview guide	Qualitative: Directed content analysis based on TAM2	5
Jensen and McKerrow (2022)	South Africa	To investigate whether an electronic decision support tool to strengthen IMCI implementation is acceptable to nurses, clinic managers and caregivers at primary care facilities in KwaZulu‐Natal, South Africa.	Hospital and Immunization centre 32 Nurses	Mixed method: Quantitative – Qualitative Data collected using: Quantitative: pre and post questionnaires. Qualitative—focus group discussions, in‐depth interviews, exit interviews.	Quantitative: Descriptive and inferential statistics Qualitative: Content analysis; manual	4
Kahouei, Daimazar, and Firouzeh (2015)	Iran	To investigate the experiences of nurses about the effect of electronic patient records (EPR) on their routine activities	Hospital 180 Nurses (112 used for analysis)	Quantitative: Cross‐sectional surveys Data collected using a questionnaire	Quantitative: Descriptive and inferential statistics	2
Kahouei, Zadeh, and Roghani (2015)	Iran	To evaluate head nurses' and supervisors' perceptions about the efficiency of the electronic patient record (EPR) system and its impact on nursing management tasks.	Hospital 316 Nurses	Quantitative: Cross‐sectional surveys Data collected using a questionnaire	Quantitative: Descriptive and inferential statistics	5
Kamil et al. (2020)	Indonesia	To explore health professionals' perception of Health‐ID (an electronic patient progress documentation)	Hospital 16 Nurses	Qualitative: Focus group discussion and interviews Data collected using interview and FGD guides	Qualitative: Thematic analysis	5
Kartika et al. (2021)	Indonesia	To understand clinicians' perception of electronic health record implementation	Outpatient setting 137 Nurses	Quantitative: Cross‐sectional surveys Data collected using a questionnaire	Quantitative: Descriptive and inferential statistics	3
Lei et al. (2023)	China	To develop a logic model for adopting electronic medication administration records (eMAR) in long‐term care facilities (LTCFs) and explore the contextual factors relevant to implementing eMAR in LTCFs.	Long‐Term Care homes 20 Nurses	Qualitative: Interviews Data collected using an interview guide	Qualitative: Thematic analysis; inductive	5
Mahdizadeh et al. (2022)	Iran	To examine Iranian nurses' attitudes toward the challenges and opportunities of using the National Electronic Health Record (NEHR) system in healthcare organizations.	Hospital 135 Nurses	Quantitative: Cross‐sectional surveys Data collected using a questionnaire adapted from Gorzin et al. (2016).	Quantitative: Descriptive and inferential statistics	3
Makeleni and Cilliers (2021)	South Africa	To investigate the critical success factors that can improve the data quality of EMRs in public healthcare institutions in the Northwest province of South Africa	Hospital and Outpatient setting 7 Nurses	Qualitative: Interviews Data collected using an interview guide	Qualitative: Content analysis	5
Özer and Şantaş (2020)	Turkey	To investigate nurses' views regarding the effect of electronic medical records on patient safety culture.	Hospital 398 Nurses	Quantitative: Cross‐sectional surveys Data collected using the Use, Quality and User Satisfaction with EMR Systems Questionnaire, developed by Otieno et al. (2007) and adapted to Turkish by Top et al. (2015).	Quantitative: Descriptive and inferential statistics	3
Peivandi et al. (2022)	Iran	To evaluate the use of online and offline speech recognition software on spelling errors in nursing reports and to compare them with errors in handwritten reports.	Hospital 70 Nurses	Quantitative: Randomized Control Trial (RCTs); Crossover study design (2 groups) Data collected using a checklist and EHR data review	Quantitative: Descriptive and inferential statistics	4
Peng et al. (2020)	China	To study the nursing risk warning from patients' electronic medical records.	Hospital 200 Nursing care records	Quantitative: Cross‐sectional retrospective surveys. Data collected using a questionnaire.	Quantitative: Descriptive and inferential statistics	5
Qin et al. (2017)	China	To investigate the development, implementation, use and impact of an intensive care information system (ICIS) in a Chinese hospital on nursing processes and outcomes.	Hospital 27 Nurses	Qualitative: Focus group discussion (FGD), Case study Data collected using: (1) a FGD guide (2) Checklist for EHR data	Qualitative: Thematic analysis	4
Selna et al. (2022)	Maldives	To investigate patient safety and challenges faced by nurses in using electronic health records (EHRs) to enhance patient safety in two large hospitals in Maldives.	Hospital 10 Nurses	Qualitative: Focus group discussion (FGD), Case study. Data collected using: (1) Semi‐structured (2) Open‐ended questionnaire	Qualitative: Thematic analysis	4
Shafiee et al. (2022)	Iran	To describe the process of designing and evaluating the content of an electronic clinical nursing documentation system (ECNDS) to provide consistent and unified reporting in this context.	Hospital 150 Nurses	Quantitative: Two rounds Delphi study and usability surveys Data collected using a questionnaire and the Delphi study data	Quantitative: Descriptive and inferential statistics	5
Sinha and Joy (2022)	India	To assess the knowledge of attitude toward and use of a Nursing Information System (NIS).	Hospital 230 Nurses	Quantitative: Prospective cross‐sectional study Data collected using a questionnaire	Quantitative: Descriptive and inferential statistics	5
Tierney et al. (2016)	Kenya	To assess whether the primary care EMR was used for all patients and visits, and to evaluate the impact of the EMR on patient flow, provider work and user satisfaction in three rural Kenyan health centres.	Outpatient setting 14 Nurses	Quantitative: Time‐motion study design and Surveys Data collected using: (1) An author‐developed questionnaire for surveys. (2) An author‐developed Time‐motion encounter form/checklist.	Quantitative: Descriptive and inferential statistics	4
Tilahun and Fritz (2015)	Ethiopia	To assess the current EMR use rate among health professionals; assess the use level of core EMR functions; determine the user satisfaction level of health professionals; and identify determinant factors of user satisfaction toward the EMR system.	Hospital 176 Nurses	Quantitative: Cross‐sectional surveys Data collected using an adapted questionnaire from literature.	Quantitative: Descriptive and inferential statistics	5
Tubaishat (2017)	Jordan	To evaluate electronic health records (EHRs), from the perspective of nurses, by examining how they use the records, their opinions on the quality of the systems, and their overall levels of satisfaction with EHRs.	Hospital 1648 Nurses	Quantitative: Cross‐sectional surveys Data collected using a questionnaire	Quantitative: Descriptive and inferential statistics	5
Tubaishat (2019)	Jordan	To explore the effect of EHRs on patient safety, as perceived by nurses	Hospital 17 Nurses	Qualitative: Interviews Data collected using an interview guide	Qualitative: Thematic analysis	5
Uzun and Cerit (2023)	Turkey	To examine the effect of digitalization on nursing practices using the lean approach	Hospital 15 Nurses	Quantitative: Cross‐sectional surveys Data collected using: (1) Activity charts and (2) Value Stream Map (VSM)	Quantitative: Current and future state value stream mapping, lean seven waste areas analysis	4
Venkateswaran et al. (2022)	Palestine	To evaluate the time spent on health information management in eRegistry clinics compared to clinics performing paper‐based documentation.	Outpatient setting 22 Nurses and midwives	Quantitative: Randomized Control Trial (RCTs); Time‐motion studies. Data collected using a customized Microsoft Access template from the US Agency for Healthcare Research and Quality	Quantitative: Descriptive and inferential statistics	5
Wang et al. (2016)	China	To develop a nursing information system (NIS) to enhance the visibility of patient risk, and to identify challenges and facilitators to the adoption of the NIS risk assessment system for nurse leaders.	Hospital 667 Nurse	Quantitative: Cross‐sectional; Pre and post survey Data collected using a questionnaire	Quantitative: Descriptive and inferential statistics	4
Yilmaztürk et al. (2023)	Turkey	To measure the effect of digitizing medical records kept in paper forms in ICUs on timesaving and paper consumption.	Hospital 428 Nursing care records 2 Nurses	Quantitative: Cross‐sectional retrospective survey; EHR data review Data collected using an Excel data form	Quantitative: Descriptive and inferential statistics	4
Zhai et al. (2022)	China	To investigate nurses' perceptions and experiences with transition to a new nursing information system (Care Direct) 2 years after its first introduction.	Hospital 20 Nurses for qualitative 324 Nurses for quantitative	Mixed method: Concurrent Qualitative—Quantitative Data collected using: (1) Qualitative—a semi‐structured interview guide. (2) Quantitative—Online survey Nursing information system use behaviour scale by Wen et al. (2017), the Clinical Nursing Information System Effectiveness Evaluation Scale by Zhao et al. (2020), and a Questionnaire on the degree and experience of participating in system development by Martikainen et al. (2014) and Martikainen et al. (2014).	Qualitative: Thematic and Content analysis. ‐ Quantitative: Descriptive and inferential statistics	5

Abbreviations: EHR, Electronic health records; EMR, Electronic medical records; FGD, Focus group discussion; HCP, Healthcare providers; LTCFs, Long‐term care facilities; MMAT, Mixed Methods Appraisal Tool (MMAT) version 2018 by Hong et al. (2018); N/A, Not applicable; QA, Quality Appraisal.

^a^ Sample included other healthcare professionals. However, the responses of nurses were identifiable and extractable.

### 4.2 Impact of Electronic Health Records on Nurses and Nursing Care

**FIGURE 3:**
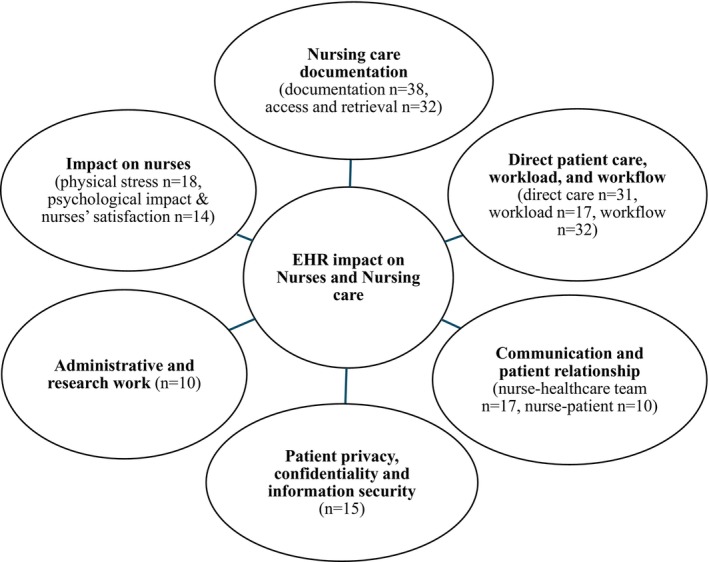
Identified Domains of EHR Impact on Nurses and Nursing Care in LMICs.

As shown in Figure 3 and Table 2, EHRs impacted nurses and nursing care in several areas, grouped under six main categories: (1) Nursing care documentation (documentation *n* = 38, 92.7%; documentation access and retrieval *n* = 32, 78.0%); (2) Direct care, workload and workflow (direct care *n* = 31, 75.6%, workload *n* = 17, 41.5%, workflow *n* = 32, 78.0%); (3) Communication (nurse‐healthcare team *n* = 17, 39.0%, nurse–patient *n* = 10, 24.4%); (4) Patient privacy, confidentiality and information security (*n* = 15, 36.6%); (5) Administrative and research work (*n* = 10, 24.4%); and (6) Impact on nurses (physical stress *n* = 18, 44.0%, psychological impact *n* = 5, 12.2% and satisfaction *n* = 9, 22.0%). The following sections present the detailed results from the included studies.

**TABLE 2:** Positive and Negative Impact of EHR use on Nurses and Nursing Care in LMICs (*n* = 41).

Category of findings	Results	
	Freq. (*n* = 41) and Percentage	Specific findings within each category
**Findings related to the impact of EHRs on nursing care**		
Nursing and patient care data documentation	38 (92.7%)	Positive impact EHReadily available, complete and up‐to‐date patient information (Arikan et al. 2022; Farshi et al. 2015; Gomes et al. 2019; Hariyati et al. 2020; Heidarizadeh et al. 2017; Jensen and McKerrow 2022). Improved documentation quality, legibility and reduced errors (Adereti and Olaogun 2019; Akhu‐Zaheya et al. 2018; Cheung and Yip 2024; Mahdizadeh et al. 2022; Shafiee et al. 2022; Sinha and Joy 2022; Tubaishat 2017). Reduction in the use of paperwork (Abed et al. 2022) Enhanced documentation processes and structure (Dolan et al. 2023; Uzun and Cerit 2023; Wang et al. 2016; Yilmaztürk et al. 2023), documentation time savings (Farshi et al. 2015; Faujdar et al. 2021; Firouzeh et al. 2017; Makeleni and Cilliers 2021; Özer and Şantaş 2020; Qin et al. 2017; Venkateswaran et al. 2022). Production of summary reports for clinical and administrative work (Galani et al. 2021; Lei et al. 2023; Peivandi et al. 2022; Tilahun and Fritz 2015).
		2 Negative impact Increased time used for documentation (Alfuqaha et al. 2022; Attafuah et al. 2022; Bei‐lei et al. 2019). Incomplete, documentation errors and poor quality of entered data (Attafuah et al. 2022; Cohen et al. 2016; Kahouei, Daimazar, and Firouzeh 2015; Kahouei, Zadeh, and Roghani 2015; Kamil et al. 2020; Tilahun and Fritz 2015; Zhai et al. 2022). Threat to patient safety data quality issues (Makeleni and Cilliers 2021; Tubaishat 2017). Restrictive or structured EHR documentation frameworks identified as unsatisfactory (Akhu‐Zaheya et al. 2018; Selna et al. 2022). Online speech recognition EHR software results in a higher number of errors compared to offline speech recognition EHR and handwritten records (Peivandi et al. 2022). Repetitive data entry in some EHRs (Farshi et al. 2015; Firouzeh et al. 2017; Galani et al. 2021).
Accessibility and ease of retrieval of patient data	32 (78.0%)	Positive impact Ease of access and retrieval of patient information (Alfuqaha et al. 2022; Arikan et al. 2022; Attafuah et al. 2022; Gomes et al. 2019; Hariyati et al. 2020; Jensen and McKerrow 2022; Kahouei, Daimazar, and Firouzeh 2015; Kahouei, Zadeh, and Roghani 2015; Kamil et al. 2020; Kartika et al. 2021; Mahdizadeh et al. 2022; Makeleni and Cilliers 2021; Özer and Şantaş 2020; Qin et al. 2017; Shafiee et al. 2022; Sinha and Joy 2022; Tubaishat 2017, 2019; Uzun and Cerit 2023; Wang et al. 2016). Efficiently stored and searchable information (Cheung and Yip 2024; Dolan et al. 2023; Faujdar et al. 2021; Firouzeh et al. 2017; Galani et al. 2021).
		2 Negative impact Difficulty navigating EHRs and accessing records (Attafuah et al. 2022) Information and data transfer disruptions from: poor system design (Alfuqaha et al. 2022; Arikan et al. 2022), slow systems (Cohen et al. 2016), and system down times (Kahouei, Daimazar, and Firouzeh 2015; Selna et al. 2022; Venkateswaran et al. 2022; Zhai et al. 2022) Fragmented information leading to use of several interfaces in the EHR system (Farshi et al. 2015; Firouzeh et al. 2017; Galani et al. 2021) Voice EHRs easily deteriorates as compared to paper documentation (Peivandi et al. 2022)
Nurse – healthcare team communication	17 (39.0%)	Positive impact Reduces the need for verbal order prescriptions (Cheung and Yip 2024) Improved communication (Hariyati et al. 2020; Jensen and McKerrow 2022; Kamil et al. 2020; Kartika et al. 2021; Lei et al. 2023; Tierney et al. 2016; Uzun and Cerit 2023; Wang et al. 2016) Better intra and inter‐department information exchange (Farshi et al. 2015)
		2 Negative impact Lack of common clinical language for healthcare team member (Alfuqaha et al. 2022; Arikan et al. 2022; Galani et al. 2021; Gomes et al. 2019). Rigid nursing documentation framework (Selna et al. 2022). Not conducive for reflecting the dynamic changes of patient conditions (Zhai et al. 2022). Altered working relations between doctors and nurses (Cohen et al. 2016; Farshi et al. 2015).
Nursing workflow and efficiency	32 (78.0%)	Positive impact Improved workflow (Abed et al. 2022; Alfuqaha et al. 2022; Attafuah et al. 2022; Faujdar et al. 2021; Jensen and McKerrow 2022; Kartika et al. 2021; Lei et al. 2023; Mahdizadeh et al. 2022; Qin et al. 2017; Shafiee et al. 2022; Sinha and Joy 2022; Tubaishat 2017; Uzun and Cerit 2023; Venkateswaran et al. 2022; Yilmaztürk et al. 2023). Easier documentation process and patient information storage (Akhu‐Zaheya et al. 2018). Reduction in documentation duplication (Faujdar et al. 2021; Wang et al. 2016) Increased productivity (Dolan et al. 2023). Improved organization of services and planning of care (Gomes et al. 2019; Heidarizadeh et al. 2017).
		2 Negative impact Longer times spent in accessing patient records resulting in patient care delays (Arikan et al. 2022; Attafuah et al. 2022; Bei‐lei et al. 2019; Dolan et al. 2023). Insufficient EHR hardware affected workflow (Özer and Şantaş 2020; Zhai et al. 2022). Increased workload and time needed for recording patient information (Dolan et al. 2023; Farshi et al. 2015; Tierney et al. 2016; Tilahun and Fritz 2015). Slow EHRs and time consuming to use EHR (Abed et al. 2022; Akhu‐Zaheya et al. 2018; Arikan et al. 2022; Bei‐lei et al. 2019; Cheung and Yip 2024; Farshi et al. 2015; Kahouei, Daimazar, and Firouzeh 2015; Kahouei, Zadeh, and Roghani 2015; Makeleni and Cilliers 2021). Difficulty accessing support during nights and weekends (Cohen et al. 2016).
Direct nursing and patient care	31 (75.6%)	Positive impact Allowed nurses more time for direct patient care (Abed et al. 2022; Arikan et al. 2022; Attafuah et al. 2022; Cohen et al. 2016; Faujdar et al. 2021; Kartika et al. 2021; Mahdizadeh et al. 2022; Makeleni and Cilliers 2021; Shafiee et al. 2022; Sinha and Joy 2022; Wang et al. 2016). Enhanced quality of care (Alfuqaha et al. 2022; Dolan et al. 2023; Faujdar et al. 2021; Gomes et al. 2019; Hariyati et al. 2020; Kartika et al. 2021; Lei et al. 2023; Tilahun and Fritz 2015; Tubaishat 2017, 2019). Improved medication administration and patient safety (Qin et al. 2017; Uzun and Cerit 2023). Improved prediction of patient deterioration (Peng et al. 2020).
		2 Negative impact Service delivery affected negatively from patient information access difficulties (Alfuqaha et al. 2022; Tierney et al. 2016). Lack of coordination among different professionals affected patient care (Galani et al. 2021; Selna et al. 2022). Non‐supportive EHRs negatively impacted care to patient (Hariyati et al. 2020; Kahouei, Daimazar, and Firouzeh 2015; Kahouei, Zadeh, and Roghani 2015; Kartika et al. 2021; Lei et al. 2023; Zhai et al. 2022).
The nurse–patient communication and relationship	10 (24.4%)	Positive impact Improved nurse–patient relationships (Farshi et al. 2015; Faujdar et al. 2021). Improved attentiveness to patients and patient interactions (Jensen and McKerrow 2022; Sinha and Joy 2022; Tubaishat 2017; Uzun and Cerit 2023; Zhai et al. 2022).
		2 Negative impact Nurses spent more time on the EHRs and computers than patients care (Abed et al. 2022; Hariyati et al. 2020). Disrupted nurse–patient communication and reduced face‐to‐face interaction (Jensen and McKerrow 2022). Disrupted therapeutic relationship and depersonalized care (Gomes et al. 2019; Sinha and Joy 2022).
Nursing workload	17 (41.5%)	Positive impact Reduced patient waiting time (Abed et al. 2022; Attafuah et al. 2022). Reduced workload (Cheung and Yip 2024; Uzun and Cerit 2023). Eliminate unnecessary movement and physical carrying of patient files (Dolan et al. 2023).
		2 Negative impact Increased patient loads (Abed et al. 2022; Arikan et al. 2022; Attafuah et al. 2022; Galani et al. 2021; Sinha and Joy 2022). Double documentation burden (EHR and paper‐based) and time‐consuming documentation processes in EHRs (Akhu‐Zaheya et al. 2018; Dolan et al. 2023; Galani et al. 2021; Jensen and McKerrow 2022; Kamil et al. 2020; Tierney et al. 2016; Zhai et al. 2022). EHR learning and adaptation difficulties adds to workload (Alfuqaha et al. 2022; Zhai et al. 2022).
Administrative and research work	10 (24.4%)	Positive impact Increased administrative oversight of nursing work (Abed et al. 2022; Farshi et al. 2015) Readily available patient care data and summary reports (Cheung and Yip 2024; Faujdar et al. 2021; Galani et al. 2021; Wang et al. 2016) Improved decision‐making and reduced infection and mortality rates (Galani et al. 2021; Qin et al. 2017) Promotes feedback from nurses and reveal their support needs to nurse managers (Abed et al. 2022; Farshi et al. 2015) Reduction in legal issues (Shafiee et al. 2022).
		2 Negative impact Some EHR systems did not support administrative work (Kahouei, Daimazar, and Firouzeh 2015; Kahouei, Zadeh, and Roghani 2015; Selna et al. 2022). Nurses feel transferring data into the EHR is not their duty (Tilahun and Fritz 2015).
Patient privacy, confidentiality, and information security	15 (36.6%)	Positive impact Improved patient privacy and security of patient information (Firouzeh et al. 2017; Peng et al. 2020; Shafiee et al. 2022; Sinha and Joy 2022; Tubaishat 2019; Wang et al. 2016). Increased patient safety culture and practices among nurses (Makeleni and Cilliers 2021; Özer and Şantaş 2020).
		2 Negative impact Easy access to patient information by unauthorized personnel (Attafuah et al. 2022; Jensen and McKerrow 2022). Increased risks for breaches of information security, privacy and confidentiality and security of patient information (Attafuah et al. 2022; Jensen and McKerrow 2022; Selna et al. 2022).
**Findings related to impact of EHRs on nurses**		
Physical stress	18 (44.0%)	Positive impact Reduced physical stress and burnout among nurses using EHRs (Abed et al. 2022; Bei‐lei et al. 2019; Dolan et al. 2023; Lei et al. 2023; Mahdizadeh et al. 2022; Sinha and Joy 2022; Venkateswaran et al. 2022; Yilmaztürk et al. 2023).
		2 Negative impact Increased physical stress and exhaustion among nurses due to: – Inadequate hardware and workstations increases workload and physical stress (Abed et al. 2022; Alfuqaha et al. 2022; Attafuah et al. 2022). – Poor system design, increased documentation burden and usability issues are physically demanding (Cheung and Yip 2024; Galani et al. 2021; Hariyati et al. 2020; Jensen and McKerrow 2022; Kamil et al. 2020; Selna et al. 2022; Zhai et al. 2022).
Psychological impact	14 (34.1%)	Positive impact Nurses were satisfied with the use of EHR systems (Hariyati et al. 2020; Kamil et al. 2020; Özer and Şantaş 2020; Shafiee et al. 2022). Generational differences in the satisfaction level: older nurses found it hard and unsatisfactory while the young ones were welcoming and very much satisfied with the EHR system (Kamil et al. 2020).
		2 Negative impact Nurses experienced anger and frustration with EHRs (Cohen et al. 2016; Dolan et al. 2023; Hariyati et al. 2020; Jensen and McKerrow 2022; Tierney et al. 2016; Tubaishat 2019). Increased mental stress from a heightened sense of nurses' work being monitored by nurse managers (Faujdar et al. 2021). Feeling embarrassed for not being able to use the EHR systems and making frequent mistakes (Kamil et al. 2020). Worry about threats to patient safety and information security (Tubaishat 2019). Dissatisfaction with the various challenges of using EHRs (Attafuah et al. 2022; Selna et al. 2022).

Abbreviations: EHR, Electronic health records; EMR, Electronic medical records.

#### 4.2.1 Impact of EHRs on Nursing Care

##### 4.2.1.1 Nursing Care Documentation, Access and Retrieval

As shown in Table 2, 38 studies highlighted the impact of EHR on nursing care documentation. The use of EHRs by nurses has significantly reduced paperwork (Abed et al. 2022), improved documentation quality and legibility (Adereti and Olaogun 2019; Akhu‐Zaheya et al. 2018; Cheung and Yip 2024; Mahdizadeh et al. 2022; Shafiee et al. 2022; Sinha and Joy 2022; Tubaishat 2017), and ensured readily available and complete patient information (Arikan et al. 2022; Gomes et al. 2019; Hariyati et al. 2020; Heidarizadeh et al. 2017; Jensen and McKerrow 2022). EHRs have also enhanced documentation processes and structure (Dolan et al. 2023; Uzun and Cerit 2023; Wang et al. 2016; Yilmaztürk et al. 2023), saved documentation time (Farshi et al. 2015; Faujdar et al. 2021; Firouzeh et al. 2017; Makeleni and Cilliers 2021; Özer and Şantaş 2020; Qin et al. 2017; Venkateswaran et al. 2022), and facilitated the production of summary reports for clinical purposes (Galani et al. 2021; Lei et al. 2023; Peivandi et al. 2022; Tilahun and Fritz 2015). For instance, in a Jordanian study, Abed et al. (2022) noted a 77.7% reduction in the use of paper‐based documentation by nurses, while Arikan et al. (2022) observed a 57.7% improvement in documentation efficiency and time savings in a Turkish study. Notably, Shafiee et al. (2022) observed a 65% reduction in legal issues for nurses and hospitals in Iran post‐EHR implementation, attributed to improved documentation of nursing care plans within a legal, ethical and professional framework.

While improved documentation processes have been reported, 17 studies noted increased documentation time with EHR use (Alfuqaha et al. 2022; Arikan et al. 2022; Attafuah et al. 2022; Bei‐lei et al. 2019). For instance, Arikan et al. (2022) observed a 42.3% increase in documentation time in a Turkish study. Issues of incomplete, inaccurate, and poor‐quality patient data entry were also reported (Attafuah et al. 2022; Cohen et al. 2016; Kahouei, Daimazar, and Firouzeh 2015; Kahouei, Zadeh, and Roghani 2015; Kamil et al. 2020; Peivandi et al. 2022; Tilahun and Fritz 2015; Zhai et al. 2022). For instance, Peivandi et al. (2022) reported continuous occurrence of documentation errors in an Iranian EHR system. Some studies identified the restrictive or structured nature of some EHR documentation platforms as inadequate for nursing needs (Akhu‐Zaheya et al. 2018; Selna et al. 2022; Zhai et al. 2022). In China, Zhai et al. (2022) found that structured EHR documentation frameworks compromised its usefulness in matching the complex and dynamic workflows of nurses.

Thirty‐two studies also documented the positive impact of EHR use on patient data accessibility and retrieval. Key benefits included easily accessible and retrievable patient information (Alfuqaha et al. 2022; Arikan et al. 2022; Attafuah et al. 2022; Gomes et al. 2019; Hariyati et al. 2020; Jensen and McKerrow 2022; Kahouei, Daimazar, and Firouzeh 2015; Kamil et al. 2020; Kartika et al. 2021; Lei et al. 2023; Mahdizadeh et al. 2022; Makeleni and Cilliers 2021; Özer and Şantaş 2020; Qin et al. 2017; Shafiee et al. 2022; Sinha and Joy 2022; Tubaishat 2017, 2019; Uzun and Cerit 2023; Wang et al. 2016), efficient storage and searchable patient data (Cheung and Yip 2024; Dolan et al. 2023; Faujdar et al. 2021; Firouzeh et al. 2017; Galani et al. 2021), and enhanced intra‐ and inter‐departmental information exchange with real‐time patient data availability (Farshi et al. 2015; Shafiee et al. 2022; Wang et al. 2016). For example, Alfuqaha et al. (2022) reported a 57.5% improvement in patient information access and retrieval in a Jordanian study. A Kenyan study made similar findings (Dolan et al. 2023).

Despite the positives, 12 studies reported negative impacts of EHRs on patient data storage, access, and retrieval. Nurses' unfamiliarity with EHR systems, inability to use them effectively, patient information and data transfer disruptions, and slow systems affected care decision‐making and direct care (Alfuqaha et al. 2022; Arikan et al. 2022; Attafuah et al. 2022; Cohen et al. 2016). Repetitive data recording and navigation between multiple user interfaces were perceived as cumbersome (Farshi et al. 2015; Firouzeh et al. 2017; Galani et al. 2021). For instance, Alfuqaha et al. (2022) reported difficult and limited access to patient records in Jordanian EHRs due to nurses' lack of necessary skills to operate the system, while in South Africa, Cohen et al. (2016) reported that slow EHR system made information retrieval difficult for nurses.

##### 4.2.1.2 Direct Patient Care, Workload, and Workflow

Thirty‐one studies also reported on direct patient care. Improved care (Abed et al. 2022; Arikan et al. 2022; Attafuah et al. 2022; Cohen et al. 2016; Mahdizadeh et al. 2022; Makeleni and Cilliers 2021; Shafiee et al. 2022; Sinha and Joy 2022; Wang et al. 2016), enhanced care quality (Alfuqaha et al. 2022; Dolan et al. 2023; Faujdar et al. 2021; Gomes et al. 2019; Hariyati et al. 2020; Kartika et al. 2021; Lei et al. 2023; Tilahun and Fritz 2015; Tubaishat 2017), better medication administration (Qin et al. 2017; Uzun and Cerit 2023), and accurate patient deterioration prediction (Peng et al. 2020) were reported. For example, Shafiee et al. (2022) reported a 92% satisfaction rate among Iranian nurses regarding the quality of care facilitated by EHRs. Tubaishat (2019) in a Jordanian study and Uzun and Cerit (2023) in Turkish study found that EHRs improved medication administration with clearer prescriptions and fewer errors. Enhanced patient safety through readily available medication information, alerts, and patient data (Lei et al. 2023; Sinha and Joy 2022; Tubaishat 2019; Uzun and Cerit 2023) was also reported. These results suggest EHRs enhanced patient care, nurse satisfaction, and medication safety while reducing errors.

While there were benefits reported, 10 studies reported negative outcomes of EHR use on direct patient care by nurses. Inaccessible EHR records (Attafuah et al. 2022; Tierney et al. 2016) and delayed access to laboratory results (Galani et al. 2021; Selna et al. 2022) on the system were found to impede healthcare delivery. There were indications that some systems did not adequately support the coordination of patient care (Galani et al. 2021; Kahouei, Daimazar, and Firouzeh 2015; Kahouei, Zadeh, and Roghani 2015; Selna et al. 2022; Zhai et al. 2022). Also, the implementation of not‐fit‐for‐purpose EHR systems (Selna et al. 2022; Tubaishat 2019), improper use and incomplete patient information were found to negatively impact patient safety in medication administration through absence of prompts and alerts (Makeleni and Cilliers 2021; Tubaishat 2019). For instance, Tierney et al. (2016) reported in a time‐motion study that Kenyan nurses spent significantly less time (80%) in direct patient care due to EHR use. Similar negative impacts on patient care delivery were reported in studies from Ghana (Attafuah et al. 2022), Iran (Firouzeh et al. 2017), and the Maldives (Selna et al. 2022). On the contrary, Farshi et al. (2015) and Venkateswaran et al. (2022) found no significant difference in patient care between paper‐based records and EHRs.

The impact of EHR implementation on nurses' workload was reported in 17 studies. Key findings included reduced patient waiting times for services (Abed et al. 2022; Attafuah et al. 2022), decreased overall workload and elimination of the need to physically transport several patient files, and the reduction of cumbersome paperwork (Abed et al. 2022; Dolan et al. 2023; Uzun and Cerit 2023). These factors collectively contributed to a diminished workload for nurses resulting from EHR adoption. For instance, Abed et al. (2022) found that EHRs reduced paperwork and documentation burden by 77.7%, significantly easing nurses' workload (56.9%) in a Jordanian study. Corroborating these findings, Dolan et al. (2023) reported in a Kenyan study that EHR usage alleviated nurses' workload, enabling them to deal with a larger number of patients. These results underscore the potential of EHRs to streamline nursing workflows and enhance efficiency in patient care delivery.

Notwithstanding the positive impact on nursing workload, 11 studies reported negative impact findings. The introduction of EHRs alongside paper‐based records in some settings led to double documentation, increased documentation burden and workload (Dolan et al. 2023; Galani et al. 2021; Jensen and McKerrow 2022; Kamil et al. 2020; Sinha and Joy 2022; Tierney et al. 2016). Further, missing patient information, recurrent documentation, and increased documentation time contributed to increased workloads (Abed et al. 2022; Akhu‐Zaheya et al. 2018; Arikan et al. 2022; Bei‐lei et al. 2019; Farshi et al. 2015; Makeleni and Cilliers 2021). For instance, nurses reported increased patient loads attributable to time savings and improved workflow from EHR use in a Jordanian (Abed et al. 2022) and Ghanaian (Attafuah et al. 2022) studies. Galani et al. (2021) reported increased workload from dual documentation in Botswana, while Sinha and Joy (2022) found that 43.3% of nurses in an Indian study perceived increased workload due to EHR use. Bei‐lei et al. (2019) noted that Chinese nurses found EHRs time‐consuming and demanding, thus contributing to physical stress. In a Turkish study (Arikan et al. 2022), 37% of nurses perceived increased workload due to EHRs.

Nursing workflow was reported in 32 studies. EHRs use led to better time management, leading to increased availability of time for direct patient care activities (Abed et al. 2022; Alfuqaha et al. 2022; Faujdar et al. 2021; Jensen and McKerrow 2022; Kartika et al. 2021; Lei et al. 2023; Mahdizadeh et al. 2022; Qin et al. 2017; Shafiee et al. 2022; Sinha and Joy 2022; Tubaishat 2017; Uzun and Cerit 2023; Venkateswaran et al. 2022; Wang et al. 2016; Yilmaztürk et al. 2023). For instance, Yilmaztürk et al. (2023) reported a daily time savings of 56.8 min per nurse in Turkey, Venkateswaran et al. (2022) observed a reduction in manual documentation time from a mean of 8.48 min to 5.50 min among Palestinian nurses, while Shafiee et al. (2022) observed an 82% increase in patient care time in Iran as a result of improved workflow and time efficiency from EHRs. Further, easily accessible patient information in EHRs improved the organization of nursing care, clinical decision‐making, and made work easy (Dolan et al. 2023; Gomes et al. 2019; Heidarizadeh et al. 2017). These findings collectively highlight the positive impact of EHRs on workflow efficiency, particularly in care planning, organization, and delivery.

Nonetheless, 17 studies reported nursing work and patient care disruptions due to the increased amount of timespent on recording and accessing patient records in EHR systems (Arikan et al. 2022; Attafuah et al. 2022; Bei‐lei et al. 2019; Cohen et al. 2016; Dolan et al. 2023; Hariyati et al. 2020; Makeleni and Cilliers 2021; Tierney et al. 2016). Workflow disruptions were mostly occasioned by insufficient hardware (Özer and Şantaş 2020; Zhai et al. 2022), increased patient loads (Dolan et al. 2023; Farshi et al. 2015; Tierney et al. 2016; Tilahun and Fritz 2015), slow EHR systems (Cheung and Yip 2024; Kahouei, Daimazar, and Firouzeh 2015; Kahouei, Zadeh, and Roghani 2015), and lack of system support (Cohen et al. 2016). However, Galani et al. (2021) found no significant change in nurses' workflow following EHR implementation.

##### 4.2.1.3 Nurse—Healthcare Team and Nurse—Patient Communication

The impact of EHR use on the nurse—healthcare team communication was reported in 17 studies. The use of EHRs was found to enhance verbal and written nurse‐healthcare team communication (Hariyati et al. 2020; Jensen and McKerrow 2022; Kamil et al. 2020; Kartika et al. 2021; Lei et al. 2023; Shafiee et al. 2022; Sinha and Joy 2022; Tierney et al. 2016; Uzun and Cerit 2023; Wang et al. 2016). Its use mitigated errors and legibility issues in communicating patient information (Cheung and Yip 2024). Improved communication between nurses and others was also reported in a Brazilian (Gomes et al. 2019) and Turkish (Uzun and Cerit 2023) studies. In a conflicting finding, however, Cheung and Yip (2024) noted that EHRs reduced the necessity for verbal communication within healthcare teams, while Tubaishat (Tubaishat 2019) observed that while communication channels existed in EHR systems in a Jordanian study, they were underutilized by bedside nurses, with only nurse managers actively using them.

Despite these benefits, nine studies reported negative outcomes of EHRs use on nurse‐healthcare team communication. Structured and rigid EHR frameworks were found to inadequately capture the dynamic nature of critical patient conditions, compromising the communication of patient status and changes to team members (Selna et al. 2022; Zhai et al. 2022). Over‐reliance on EHRs at the expense of verbal communication (Jensen and McKerrow 2022) and the lack of a common language in some EHR systems hindered information flow and team communication (Alfuqaha et al. 2022; Arikan et al. 2022; Galani et al. 2021; Gomes et al. 2019). However, while EHRs altered working relations between nurses and other healthcare workers, some studies found no significant difference in team communication (Cohen et al. 2016; Farshi et al. 2015).

The therapeutic nurse–patient communication and relationship was addressed in 10 studies. Positive outcomes were noted in eight studies, where enhanced nurse–patient engagement, interaction and improved therapeutic relationships were reported (Farshi et al. 2015; Faujdar et al. 2021; Jensen and McKerrow 2022; Sinha and Joy 2022; Tubaishat 2017; Uzun and Cerit 2023; Zhai et al. 2022). For instance, an Iranian study, Farshi et al. (2015) found that EHRs optimized existing nurse–patient relationships, while Jensen and McKerrow (2022) reported improved nurse attentiveness and increased patient interactions following the implementation of an EHR system in South Africa.

However, five studies identified a negative impact of EHRs on nurse–patient interactions. For instance, Jensen and McKerrow (2022) noted that nurses in South Africa reported concerns about how an over‐reliance on EHR systems disrupted their communication with patients. A Brazilian (Gomes et al. 2019) and an Indonesian (Hariyati et al. 2020) qualitative studies reported that nurses perceived EHR use as negatively impacting nurse–patient interactions and therapeutic relationships. In Jordan, Abed et al. (2022) observed that nurses spent more time on EHR devices than on patient care and interaction, compromising the therapeutic relationship. Sinha and Joy (2022) reported that nurses in India perceived EHR use as leading to more depersonalized care due to increased attention to the EHR system and computers at the expense of patient interaction.

##### 4.2.1.4 Patient Privacy, Confidentiality, and Information Security

Fifteen studies addressed the impact of EHRs on patient confidentiality, privacy, and information security. EHRs improved the privacy of patient information (Firouzeh et al. 2017; Peng et al. 2020; Shafiee et al. 2022; Sinha and Joy 2022; Tubaishat 2019; Wang et al. 2016). EHRs use increased patient safety culture and practices among nurses (Makeleni and Cilliers 2021; Özer and Şantaş 2020). These results suggest that EHRs create a safer, more secure healthcare environment by providing comprehensive, secure, and accessible patient information to nurses. However, concerns were raised in seven studies about patient privacy, confidentiality, and information security in relation to EHR use. Potential breaches in confidentiality and security of patient information were attributed to unauthorized access and the use of personal devices by nurses to access EHR systems in Ghana (Attafuah et al. 2022) and the communication of vital patient information via social media in Maldives (Selna et al. 2022).

##### 4.2.1.5 Administrative and Research Work

Ten studies reported on the impact of EHR use on nursing administrative and research work. Improvement in documentation completeness, increased administrative oversight of nursing work, and the availability of summary reports support nursing audits, research, and administrative work for improved patient care and ward management (Abed et al. 2022; Cheung and Yip 2024; Faujdar et al. 2021; Galani et al. 2021; Gomes et al. 2019; Mahdizadeh et al. 2022; Qin et al. 2017; Tilahun and Fritz 2015; Wang et al. 2016). For example, in Jordan, Abed et al. (2022) reported that EHR use facilitated feedback from nurses to nursing administrators, enabling better support for bedside nurses. Tilahun and Fritz (2015) noted that Ethiopian nurses found EHRs useful for generating patient care summary reports for clinical, administrative, and research purposes, while Brazilian nurses appreciated how EHRs facilitated their research efforts through improved data accessibility (Gomes et al. 2019). Wang et al. (2016) reported that EHR use in China enhanced data collection for research and evaluation.

Three studies, however, identified negative findings regarding the impact of EHRs on nursing administration and research. These studies indicated that the EHR systems failed to meet the information requirements necessary for effective administrative duties. Two studies in Iran by Kahouei, Daimazar, and Firouzeh (2015) and Kahouei, Zadeh, and Roghani (2015), for instance, found that nursing leaders and supervisors perceived inadequate support from EHR systems for their administrative and management tasks. In a Maldivian study, Selna et al. (2022) reported that nurses found the EHR system to be not‐so‐user‐friendly and inadequate in supporting their record‐keeping responsibilities, consequently affecting administrative and research work.

#### 4.2.2 Impact of EHRs on Nurses

##### 4.2.2.1 Psychological Impact and Nurses' Satisfaction

Fourteen studies reported on the psychological effects of the use of EHRs and the satisfaction level of nurses with these systems. Nurses were highly satisfied with the EHR systems (Hariyati et al. 2020; Kamil et al. 2020; Özer and Şantaş 2020). However, Kamil et al. (2020) noted generational differences in an Indonesian study, where older nurses reported greater difficulty with EHRs and expressed lower satisfaction, while their younger colleagues were more receptive and satisfied. An Iranian study by Shafiee et al. (2022) reported high nurse satisfaction (92%) with the quality of care, system simplicity, fluency and security provided by EHRs. Hariyati et al. (2020) found that while Indonesian nurses initially expressed frustration with EHRs, their satisfaction improved as they became more familiar with the system. In Turkey, Özer and Şantaş (2020) observed high EHR satisfaction among nurses, which positively influenced their perceptions of safety. These findings suggest that despite initial challenges and generational variations, nurses find EHRs beneficial and satisfying once they adapt to the system.

Five studies reported on the psychological effects of the use of EHRs on nurses. The studies reported the feelings of worry, frustration, embarrassment, and dissatisfaction among nurses using EHRs, which were attributed to system slowness, connectivity issues, incomplete records, and double documentation practices (Cohen et al. 2016; Faujdar et al. 2021; Hariyati et al. 2020; Jensen and McKerrow 2022; Tubaishat 2019). For instance, in Indonesia, nurses found EHR use to be frustrating and time‐consuming (Hariyati et al. 2020). Tubaishat (2019) reported that perceived threats to patient safety from EHR use caused significant worry and dissatisfaction among nurses in a Jordanian study, while in Iran, Faujdar et al. (2021) observed that nurses experienced heightened pressure, with a feeling of being ‘surveilled’ due to the easy scrutiny of their EHR work records.

##### 4.2.2.2 Physical Impact on Nurses

Eighteen studies addressed the impact of EHR implementation on nurses. The reduction in workload, manual documentation burden, data capture redundancy, documentation errors and medication administration errors in some studies reportedly led to reduced physical stress and burnout among nurses using EHRs (Abed et al. 2022; Bei‐lei et al. 2019; Dolan et al. 2023; Lei et al. 2023; Mahdizadeh et al. 2022; Qin et al. 2017; Sinha and Joy 2022; Venkateswaran et al. 2022; Yilmaztürk et al. 2023). For instance, in a Jordanian study, Abed et al. (2022) found that 77.7% of nurses reported reduced physical burden due to the elimination of paper‐based documentation, a finding corroborated by Dolan et al. (2023) in Kenya. Lei et al. (2023) noted decreased workload and the capture of redundant patient data, contributing to reduced physical stress. These findings suggest that EHR adoption alleviates some physical burden and stress associated with traditional paper‐based documentation methods for nurses.

However, 15 studies reported contrary findings, where EHR use led to increased physical stress among nurses (Abed et al. 2022; Alfuqaha et al. 2022; Bei‐lei et al. 2019; Heidarizadeh et al. 2017). This stress was primarily attributed to insufficient hardware, which led to work accumulation and physical exhaustion, documentation burden, increased patient volume, difficulties in transitioning to and navigating the EHR system (Cheung and Yip 2024; Cohen et al. 2016; Dolan et al. 2023; Galani et al. 2021; Gomes et al. 2019; Hariyati et al. 2020; Selna et al. 2022; Tilahun and Fritz 2015; Zhai et al. 2022). For instance, Cheung and Yip (2024) reported that Chinese nurses experienced increased physical burden and stress due to double documentation, increased patient volumes, and technical challenges associated with EHRs. Studies in Jordan (Akhu‐Zaheya et al. 2018), China (Bei‐lei et al. 2019), and Botswana (Galani et al. 2021) also found that increased workload and time‐consuming documentation processes contributed to nurses' physical stress.

## 5 Discussion

The findings of this scoping review highlight both benefits and unintended consequences of the use of EHR by nurses for nursing and patient care in LMICs. While EHRs have facilitated documentation efficiency, data accessibility, and workflow improvements, they have also introduced challenges such as increased nursing workload, usability concerns, and persistent data quality issues. Notably, research on EHRs in LMICs remains limited, with little work conducted in Africa, despite the region's increasing adoption of these systems. Additionally, while EHRs have improved communication and administrative processes, they have also raised concerns about security, privacy, and psychological stress among nurses. These contrasting outcomes suggest that while EHRs hold significant potential to transform nursing care, their implementation must be carefully managed to maximize benefits while mitigating unintended negative consequences. The following sections discuss the implications of the review results.

### 5.1 Limited EHR Research and LMICs Regional Disparities

This review revealed limited research on EHR impact on nurses and nursing in LMICs, with only 41 studies examining the topic in the last decade. While there has been a slight increase recently, the overall publication numbers remain low compared to high‐income countries where similar reviews identified between 120 to over 700 studies (Jedwab et al. 2019; Krick et al. 2019). Sub‐Saharan African countries were among the least represented with 9 publications between 2015 to 2024, despite the increasing EHR adoption rates (Kumar and Mostafa 2020). The regional imbalance observed, especially between the Middle‐Eastern and Sub‐Saharan countries (18 versus 9 of 41 papers), likely reflects underlying differences in digital health infrastructure, financing and research capacity across these regions (Ndung'u and Signé 2020; World Bank Group 2024b). Several of the included African studies highlight persistent structural constraints such as limited technology access, inadequate hardware, and system downtime (Attafuah et al. 2022; Cohen et al. 2016; Makeleni and Cilliers 2021; Tilahun and Fritz 2015), which complicate both implementation and sustained use of EHRs, thereby limiting opportunities to conduct and publish rigorous studies focused on nurses and nursing care.

By contrast, Middle Eastern and some Asian LMICs represented (Iran, Turkey and China) have more established EHR infrastructures and a stronger track record of health informatics research, including multiple studies examining documentation quality, workflow, usability and nurses' perceptions (Mahdizadeh et al. 2022; Özer and Şantaş 2020; Shafiee et al. 2022; Zhai et al. 2022). Addressing this imbalance will require targeted investment in robust digital infrastructure, sustained support for interoperable EHR platforms and capacity‐building for nurse‐led digital health and health informatics research in Sub‐Saharan African settings, to ensure that future evidence more equitably reflects the diversity of LMIC nursing contexts (Akanbi et al. 2012; Mugauri et al. 2025; Osman et al. 2025; World Bank Group 2024b).

### 5.2 Gaps in Theory Use and Instrument Validation

While theories and frameworks contribute to methodological rigour by providing a structured approach to study design and a lens for interpreting findings within a broader context (Wynn and Clarkson 2024), our synthesis revealed notable gaps in this regard. More than half of the included studies (63%) did not report using any explicit theory or model to guide variable selection, hypothesized relationships or interpretation of results. Moreover, 30 studies failed to clearly report the validation status of the instruments used. The absence of theory‐driven frameworks and psychometrically sound measures limits the conceptual coherence of these studies, makes it difficult to compare findings across contexts and systems, and constrains the extent to which results can be confidently generalized beyond a single institution or country (Polit and Beck 2014; Swan et al. 2023; Wyatt 2019). In under‐resourced LMIC settings, particularly in underrepresented regions such as Sub‐Saharan Africa, this is especially concerning, as contextually grounded, theory‐informed and measurement‐robust research is needed to move toward understanding of how EHR use shapes nurses' work, well‐being and care processes in ways that can reliably inform practice, policy and future research.

### 5.3 Impact of EHRs on Nurses and Nursing Practice

The findings underscore the multifaceted impact of EHRs on nursing care documentation, with 92.7% of the 41 papers touching on documentation. The evidence revealed a strong positive trend toward reduction in the use of paper‐based charts (Abed et al. 2022), enhanced patient data legibility and quality, and ensuring accessible complete patient data (Adereti and Olaogun 2019; Akhu‐Zaheya et al. 2018; Arikan et al. 2022; Cheung and Yip 2024; Hariyati et al. 2020). It supported efficient report generation for clinical use, with time efficiency gains, and reduction in legal issues (Arikan et al. 2022; Galani et al. 2021; Lei et al. 2023; Shafiee et al. 2022). These findings align with evidence from high‐income countries where the impact of EHRs on nursing practice has been extensively examined (Huter et al. 2020; Shapiro and Kamal 2022; Whitt et al. 2024). Prior research indicates that EHR systems enhance documentation legibility, facilitating the provision of real‐time access to patient information, thereby supporting efficient clinical decision‐making and improving the overall quality of care (Huter et al. 2020; McBride et al. 2023). Collectively, these result in reducing the burden of manual documentation and enable nurses to allocate more time to direct patient care.

Despite these documentation benefits, concerns regarding documentation quality including inaccuracies and incomplete records remain following the adoption of EHRs in LMICs, with several studies identifying drawbacks. Some studies reported increased documentation time following the adoption of EHRs, with Arikan et al. (2022) for instance reporting a 42.3% increase. Factors such as system complexity, poor interface design, and limited user training have been identified as contributory factors in previous studies (Baumann et al. 2018; Olakotan et al. 2025). Additionally, incomplete or inaccurate entries remain common (Kamil et al. 2020; Peivandi et al. 2022), indicating that digital systems alone do not guarantee documentation quality. The rigidity of some EHR fields for documentation prevented nurses capturing nuanced clinical observations (Akhu‐Zaheya et al. 2018; Zhai et al. 2022), thus failing to align with nurses' dynamic workflows and compromising usability and professional judgement. These issues are critical and warrant further investigations in such LMICs settings, as they have the potential to negatively impact the sustained use of EHRs, documentation quality, interprofessional communication, patient safety, and care outcomes (Fraczkowski et al. 2020).

The findings also showed that EHRs use led to improved direct patient care, workflow efficiency, as well as quicker clinical decision‐making (Nguyen et al. 2024). However, the effectiveness of EHRs is highly dependent on system design and usability. While some studies report that well‐integrated EHRs led to reduced nursing workload and enhance efficiency, others highlight increased workload and care delays resulting from cumbersome data entry processes and dual documentation (paper‐based and EHR) (Kaihlanen et al. 2021). These contrasting findings suggest that the benefits of EHRs are not universally realized, underscoring the need for continuous system assessment and optimization to ensure they support, rather than hinder, nursing practice. Implementing user‐centred design principles and integrating ongoing feedback from nurses could enhance system usability, resulting in improved quality of patient care (Chu, Conway, et al. 2022; Chu, Ronquillo, et al. 2022; Dykes and Chu 2021).

Enhanced nurse‐healthcare team communication and inter‐professional collaboration in the care of patients through enhanced access to patient information in EHRs were identified. This led to better coordinated care and improved patient outcomes in EHRs (Misto et al. 2019). Despite these benefits, the findings revealed that EHR use led to reduced face‐to‐face interactions, resulting in depersonalized care. Balancing digital documentation with meaningful nurse–patient connections is essential for care and sustained EHR use (Forde‐Johnston et al. 2023).

The vulnerability of some EHRs to unauthorized access to patient information underscores the necessity for robust security measures and raises concern among nurses about patient privacy, confidentiality and data security (Pérez‐Martí et al. 2022). These findings highlight the critical need for healthcare organizations to implement stringent protocols and technological safeguards to protect sensitive patient data and maintain compliance with professional and regulatory standards. Further, EHR system usability issues and insufficient informatics skills among nurses may compromise patient care, highlighting the need for continuous training and system optimization (Jedwab et al. 2022).

EHRs have improved administrative oversight, administrative decision‐making, nursing audits, and research by improving data availability and accessibility. However, unresolved usability and documentation quality challenges continue to compromise these functions of nursing, particularly affecting nursing research and administrative tasks (Chishtie et al. 2023; Krick 2021; Laukvik et al. 2024). This underscores the need for improved investment in fit‐for‐nursing‐purpose EHRs, nursing informatics training and continuous evaluation of EHR systems to support nursing administrative and research functions (Ndung'u and Signé 2020; Osman et al. 2025).

Our synthesis revealed that nurses in LMICs experienced stress, frustration, and burnout from using EHRs and other digital health tools. Termed as “technostress” (McBride et al. 2023), it is deeply embedded within the wider healthcare workforce, technological and resource constraints in LMICs, rather than arising solely from system usability problems (Mugauri et al. 2025). Multiple studies show that EHR‐related technostress constraints such as increased documentation burden, learning new systems and managing frequent technical issues were layered onto pre‐existing context issues such as understaffing, high patient–nurse ratios and limited informatics support (Abed et al. 2022; Attafuah et al. 2022; Bibi et al. 2023; Boakye‐Agyemang 2022; Galani et al. 2021). In a particular context, time savings or efficiency gains from EHRs were unexpectedly offset by increased patient loads, effectively negating the gains, thereby contributing to fatigue, dissatisfaction, frustration and the ‘fear’ that the systems were for monitoring productivity rather than supporting care (Attafuah et al. 2022; Faujdar et al. 2021). This dynamic is consistent with broader literature showing that technostress, burnout and turnover intention are more likely when digital transformations occur in environments characterized by high workload, staffing shortages and inadequate training or absence of local troubleshooting capacity (Demir and Erigüç 2025; Heponiemi et al. 2021; Kopuz et al. 2025; Sommovigo et al. 2023; Wirth et al. 2024).

The divergence between anticipated efficiencies from EHR implementation and nurses' reported experiences of documentation burden and technostress in LMICs suggests that technical performance alone is insufficient; digital health tools need to be designed and implemented in ways that fit nurses' clinical workflows and support, rather than disrupt, day‐to‐day care processes (Chu, Ronquillo, et al. 2022; Iannucci and Chu 2025; Irani and Chu 2022). Mitigating technostress in LMICs will also require adequate staffing, protected time for documentation and sustained investment in nurses' digital literacy, and on‐site informatics support (Heponiemi et al. 2021; Wirth et al. 2024). Therefore, integrating the nursing perspective into EHR design, implementation, and optimization is essential to ensure these systems support clinical workflows and compassionate patient‐centred care.

### 5.4 Implication for Nursing Practice, Education, Research, and Policy

The findings of this review have several implications for nursing and health systems in LMICs. For *nursing* *practice*, EHRs were associated with clearer documentation structures, improved information access, and better coordination with other healthcare professionals, but also with increased documentation burden, workflow disruptions, technostress, and episodes of depersonalized care. Persistent problems with incomplete, inaccurate or overly rigid documentation fields, as well as dual paper–EHR charting, indicate that digitalization alone does not guarantee documentation quality or safer care. Nurse leaders should therefore focus on user‐centred configuration of EHRs, adequate hardware and connectivity, and local optimization of nursing workflows, while actively monitoring both documentation quality and nurse–patient interaction to ensure that EHRs support person‐centred care rather than displacing it.

For *nursing* *education*, the review highlights recurrent concerns about documentation errors, incompleteness, double documentation, and difficulty navigating EHRs. These point to substantial gaps in clinical record‐keeping and informatics training for nurses in LMICs, which is consistent with research about nursing informatics education (Chu et al. 2025). Strengthening pre‐service and in‐service curricula in health informatics, covering EHR documentation, data quality, privacy and security, workflow integration, and the critical use of EHR data for clinical decision‐making is essential for improving documentation practices and sustaining high‐quality EHR use in nursing. This can draw on growing evidence from documentation integrity and interventions across nursing and other healthcare professional groups (Emekli et al. 2025; Hardido et al. 2023; Sanderson et al. 2025).

In terms of *nursing* *research*, the small number of primary studies from LMICs, particularly the under‐representation of Sub‐Saharan Africa, and the limited use of explicit theoretical frameworks and validated instruments reveal important gaps. Future studies should include theory‐informed, psychometrically robust, longitudinal and mixed‐methods studies that examine not only documentation quality, but also workload, technostress and burnout, nurse–patient interaction, privacy and security incidents and administrative and research uses of EHR data by nurses. Evaluating nurse‐focused educational, organizational and technological challenges and facilitators across diverse LMIC contexts will also be essential to building a transferable evidence base.

Finally, for *nursing* *policy*, regional disparities in the evidence base and the persistence of infrastructure and workforce constraints underscore the importance of policies that couple EHR implementation with investments in clinical informatics education, protected training times, appropriate staffing and governance frameworks for data quality, privacy and security. Involving nurses and other front‐line healthcare professionals in national and institutional digital health strategies, including the design, implementation, optimization and evaluation of EHR systems, will be essential to ensure that these technologies are fit for nursing practice, are sustainable in resource‐constrained settings, and contribute to equitable person‐centred nursing care in LMICs.

### 5.5 Strengths and Limitations

This scoping review contributes to the existing body of literature on digital health technology and nursing by examining the impact of EHR use on nurses and nursing care in LMICs, thereby adding to the ongoing discussion in this area. It followed the JBI scoping review methodology and reported its findings in accordance with PRISMA‐ScR guidelines. The search strategy was designed with the help of an experienced health sciences librarian. Two independent reviewers undertook study screening and data extraction.

Limiting the review to English‐language publications may have disproportionately excluded studies published in other languages. Further, the name and type of EHRs were not examined. Also, the technical details or description of the EHRs were not provided, so we are unable to extrapolate the findings to any specific EHR vendor. The included studies were limited to LMICs, and as such, findings may not be generalizable to all contexts. While we conducted a critical appraisal, it did not affect the inclusion of studies.

## 6 Conclusion

This scoping review provides critical insights into the complex interplay between EHRs and nursing in the context of LMICs. The review presents a comprehensive synthesis of evidence from primary research on the impacts of EHRs on nurses and nursing care in these resource‐limited settings in the last decade, addressing a critical gap in nursing and health literature often dominated by evidence from high‐income settings. The findings revealed that the benefits of EHRs in nursing are not universally realized in this region, as some important unintended consequences of this digital platform were reported.

While there were reports of enhanced nursing care through improved workflow efficiency, streamlined documentation processes, and better communication of patient information within healthcare teams, these benefits were, however, counterbalanced by negative unintended consequences such as increased workload, usability issues, persistent documentation quality concerns, patient information security risks, and depersonalization of care. The findings therefore emphasize the multifaceted nature of the impact of this digital health innovation on nurses and nursing care in the low‐resource settings of LMICs. The results also highlight the need for more robust studies that incorporate both validated measures and theoretical models to better understand the full impact of EHRs on nurses and nursing care and provide evidence to guide policy and practice.

## Author Contributions

Wahab Osman: conceptualization, methodology, investigation, data curation, visualization, formal analysis, writing – original draft preparation; Stephanie Asah‐Ofori: data curation, writing – editing, visualization; Alexandra Harris: supervision, writing – review and editing, validation; Vida N. Yakong: supervision, writing – review and editing, validation; Kimberley Widger: supervision, writing – review and editing, validation; and Charlene H. Chu: supervision, data curation, writing – review and editing, validation.

## Funding

This work is supported by funding from the PhD in Nursing Science Program Funding of the Lawrence Bloomberg Faculty of Nursing, University of Toronto and the Canada Graduate Research Scholarship ‐ Doctoral Award (CGRS‐D) of the Canadian Institutes of Health Research (CIHR) (Award number: 202511DTA‐577714‐432001).

## Ethics Statement

The authors have nothing to report.

## Conflicts of Interest

The authors declare no conflicts of interest.

## Supporting information


**Appendix S1:** is the literature search strategy for the databases searched.

**Appendix S2:** is the MMAT quality appraisal scores of the included studies.

**Appendix S3:** is the PRISMA‐ScR checklist of the manuscript.

**Appendix S4:** contains the theories and theoretical frameworks used by the included studies.

## Data Availability

All data used for this review have been reported in the paper and also captured in the Supporting Information.
