# Supplementary material for: The Impact of Electronic Health Records on Nurses and Nursing Care in Low‐ and Middle‐Income Countries: A Scoping Review
Source: Nurs Open. 2026 Jun 25;13(7):e70649. doi: 10.1002/nop2.70649 (PMC13303348; doi:10.1002/nop2.70649)
Supplement: Supplementary file 1 — Appendix S1: is the literature search strategy for the databases searched. [file NOP2-13-e70649-s004.docx]

# Supplementary Material A: Search Strategy for Scoping Literature Review

**Databases, Search Terms and Combinations**

1. **Ovid MEDLINE**

**and**

1. **Ovid EMBASE:**

- **Electronic health records:** (electronic or computer* or digital*) AND (“health record*” or “medical record*” or “nursing record*” or “nursing document*” or “document*” or “patient record*” or “in-patient record*” or “inpatient record*”)
- **Nursing care:** (Nurs* or “Nursing care”)
- **Lower and Middle Income Countries:** (“Low- and middle-income countries” or “low* and middle income countries” or LMICs)

(Afghanistan or Albania or Algeria or Angola or Argentina or Armenia or Azerbaijan or Bangladesh or Belarus or Belize or Benin or Bhutan or Bolivia or "Bosnia and Herzegovina" or Botswana or Brazil or Bulgaria or "Burkina Faso" or Burundi or "Cabo Verde" or "Cape Verde" or Cambodia or Cameroon or "Central African Republic" or Chad or China or Colombia or Comoros or Congo or "Costa Rica" or "Côte d'Ivoire" or "Ivory Coast" or Cuba or Djibouti or Dominica or "Dominican Republic" or Ecuador or Egypt or "El Salvador" or "Equatorial Guinea" or Eritrea or Eswatini or Ethiopia or Fiji or Gabon or Gambia or Georgia or Ghana or Grenada or Guatemala or Guinea or "Guinea Bissau" or Haiti or Honduras or India or Indonesia or Iran or Iraq or Jamaica or Jordan or Kazakhstan or Kenya or Kiribati or Korea or Kosovo or Kyrgyz or Kyrgyzstan or Lao or Lebanon or Lesotho or Liberia or Libya or Madagascar or Malawi or Malaysia or Maldives or Mali or "Marshall Islands" or Mauritania or Mauritius or Mexico or Micronesia or Moldova or Mongolia or Montenegro or Morocco or Mozambique or Myanmar or Namibia or Nepal or Nicaragua or Niger or Nigeria or "North Macedonia" or Pakistan or Palau or Palestine or "Papua New Guinea" or Paraguay or Peru or Philippines or Russia or Rwanda or Samoa or "Sao Tome and Principe" or Senegal or Serbia or "Sierra Leone" or "Solomon Islands" or Somalia or "South Africa" or "South Sudan" or "Sri Lanka" or "St. Lucia" or "St. Vincent and the Grenadines" or Sudan or Suriname or Syria or Tajikistan or Tanzania or Thailand or "Timor-Leste" or Togo or Tonga or Tunisia or Turkiye or Turkey or Turkmenistan or Tuvalu or Uganda or Ukraine or Uzbekistan or Vanuatu or Venezuela or Vietnam or Yemen or Zambia or Zimbabwe)

1. **CINAHL**

- **Electronic health records:** TX ( (electronic or computer* or digital*) N2 (health record* or medical record* or nursing record* or nursing document* or document* or patient record* or in-patient record* or inpatient record*) ) OR TI ( (electronic or computer* or digital*) N2 (health record* or medical record* or nursing record* or nursing document* or document* or patient record* or in-patient record* or inpatient record*) ) OR AB ( (electronic or computer* or digital*) N2 (health record* or medical record* or nursing record* or nursing document* or document* or patient record* or in-patient record* or inpatient record*) )
- **Nursing Care:** TX ( Nurs* or Nursing care or Nurses ) OR TI ( Nurs* or Nursing care or Nurses ) OR AB ( Nurs* or Nursing care or Nurses )
- **Lower and Middle Income Countries:** TX ( Low* and middle income countries ) OR TI ( Low* and middle income countries ) OR AB ( Low* and middle income countries )

TX ( Afghanistan or Albania or Algeria or Angola or Argentina or Armenia or Azerbaijan or Bangladesh or Belarus or Belize or Benin or Bhutan or Bolivia or "Bosnia and Herzegovina" or Botswana or Brazil or Bulgaria or "Burkina Faso" or Burundi or "Cabo Verde" or "Cape Verde" or Cambodia or Cameroon or "Central African Republic" or Chad or China or Colombia or Comoros or Congo or "Costa Rica" or "Côte d'Ivoire" or "Ivory Coast" or Cuba or Djibouti or Dominica or "Dominican Republic" or Ecuador or Egypt or "El Salvador" or "Equatorial Guinea" or Eritrea or Eswatini or Ethiopia or Fiji or Gabon or Gambia or Georgia or Ghana or Grenada or Guatemala or Guinea or "Guinea Bissau" or Haiti or Honduras or India or Indonesia or Iran or Iraq or Jamaica or Jordan or Kazakhstan or Kenya or Kiribati or Korea or Kosovo or Kyrgyz or Kyrgyzstan or Lao or Lebanon or Lesotho or Liberia or Libya or Madagascar or Malawi or Malaysia or Maldives or Mali or "Marshall Islands" or Mauritania or Mauritius or Mexico or Micronesia or Moldova or Mongolia or Montenegro or Morocco or Mozambique or Myanmar or Namibia or Nepal or Nicaragua or Niger or Nigeria or "North Macedonia" or Pakistan or Palau or Palestine or "Papua New Guinea" or Paraguay or Peru or Philippines or Russia or Rwanda or Samoa or "Sao Tome and Principe" or Senegal or Serbia or "Sierra Leone" or "Solomon Islands" or Somalia or "South Africa" or "South Sudan" or "Sri Lanka" or "St. Lucia" or "St. Vincent and the Grenadines" or Sudan or Suriname or Syria or Tajikistan or Tanzania or Thailand or "Timor-Leste" or Togo or Tonga or Tunisia or Turkiye or Turkey or Turkmenistan or Tuvalu or Uganda or Ukraine or Uzbekistan or Vanuatu or Venezuela or Vietnam or Yemen or Zambia or Zimbabwe ) OR TI ( Afghanistan or Albania or Algeria or Angola or Argentina or Armenia or Azerbaijan or Bangladesh or Belarus or Belize or Benin or Bhutan or Bolivia or "Bosnia and Herzegovina" or Botswana or Brazil or Bulgaria or "Burkina Faso" or Burundi or "Cabo Verde" or "Cape Verde" or Cambodia or Cameroon or "Central African Republic" or Chad or China or Colombia or Comoros or Congo or "Costa Rica" or "Côte d'Ivoire" or "Ivory Coast" or Cuba or Djibouti or Dominica or "Dominican Republic" or Ecuador or Egypt or "El Salvador" or "Equatorial Guinea" or Eritrea or Eswatini or Ethiopia or Fiji or Gabon or Gambia or Georgia or Ghana or Grenada or Guatemala or Guinea or "Guinea Bissau" or Haiti or Honduras or India or Indonesia or Iran or Iraq or Jamaica or Jordan or Kazakhstan or Kenya or Kiribati or Korea or Kosovo or Kyrgyz or Kyrgyzstan or Lao or Lebanon or Lesotho or Liberia or Libya or Madagascar or Malawi or Malaysia or Maldives or Mali or "Marshall Islands" or Mauritania or Mauritius or Mexico or Micronesia or Moldova or Mongolia or Montenegro or Morocco or Mozambique or Myanmar or Namibia or Nepal or Nicaragua or Niger or Nigeria or "North Macedonia" or Pakistan or Palau or Palestine or "Papua New Guinea" or Paraguay or Peru or Philippines or Russia or Rwanda or Samoa or "Sao Tome and Principe" or Senegal or Serbia or "Sierra Leone" or "Solomon Islands" or Somalia or "South Africa" or "South Sudan" or "Sri Lanka" or "St. Lucia" or "St. Vincent and the Grenadines" or Sudan or Suriname or Syria or Tajikistan or Tanzania or Thailand or "Timor-Leste" or Togo or Tonga or Tunisia or Turkiye or Turkey or Turkmenistan or Tuvalu or Uganda or Ukraine or Uzbekistan or Vanuatu or Venezuela or Vietnam or Yemen or Zambia or Zimbabwe ) OR AB ( Afghanistan or Albania or Algeria or Angola or Argentina or Armenia or Azerbaijan or Bangladesh or Belarus or Belize or Benin or Bhutan or Bolivia or "Bosnia and Herzegovina" or Botswana or Brazil or Bulgaria or "Burkina Faso" or Burundi or "Cabo Verde" or "Cape Verde" or Cambodia or Cameroon or "Central African Republic" or Chad or China or Colombia or Comoros or Congo or "Costa Rica" or "Côte d'Ivoire" or "Ivory Coast" or Cuba or Djibouti or Dominica or "Dominican Republic" or Ecuador or Egypt or "El Salvador" or "Equatorial Guinea" or Eritrea or Eswatini or Ethiopia or Fiji or Gabon or Gambia or Georgia or Ghana or Grenada or Guatemala or Guinea or "Guinea Bissau" or Haiti or Honduras or India or Indonesia or Iran or Iraq or Jamaica or Jordan or Kazakhstan or Kenya or Kiribati or Korea or Kosovo or Kyrgyz or Kyrgyzstan or Lao or Lebanon or Lesotho or Liberia or Libya or Madagascar or Malawi or Malaysia or Maldives or Mali or "Marshall Islands" or Mauritania or Mauritius or Mexico or Micronesia or Moldova or Mongolia or Montenegro or Morocco or Mozambique or Myanmar or Namibia or Nepal or Nicaragua or Niger or Nigeria or "North Macedonia" or Pakistan or Palau or Palestine or "Papua New Guinea" or Paraguay or Peru or Philippines or Russia or Rwanda or Samoa or "Sao Tome and Principe" or Senegal or Serbia or "Sierra Leone" or "Solomon Islands" or Somalia or "South Africa" or "South Sudan" or "Sri Lanka" or "St. Lucia" or "St. Vincent and the Grenadines" or Sudan or Suriname or Syria or Tajikistan or Tanzania or Thailand or "Timor-Leste" or Togo or Tonga or Tunisia or Turkiye or Turkey or Turkmenistan or Tuvalu or Uganda or Ukraine or Uzbekistan or Vanuatu or Venezuela or Vietnam or Yemen or Zambia or Zimbabwe )

1. **WEB OF SCIENCE**

- **Electronic Health Records:** (ALL=("electronic or computer*" or "digital*")) AND ALL=("health record*" or "medical record*" or "nursing record*" or "nursing document*" or "document*" or "patient record*" or "in-patient record*" or "inpatient record*")
- **Nursing care:** (ALL=("Nursing care")) OR ALL=("Nurs*")
- **LMICs:** (((((ALL=("Low- and middle-income countries")) OR ALL=("Lower and middle income countries")) OR ALL=("Low and middle income countries")) OR ALL=(LMICs)) OR ALL=("Developing Countries")) OR ALL=("middle income countries")

ALL=(Burundi or "Cabo Verde" or "Cape Verde" or Cambodia or Cameroon or "Central African Republic" or Chad or China or Colombia or Comoros or Congo or "Costa Rica" or "Côte d'Ivoire" or "Ivory Coast" or Cuba or Djibouti or Dominica or "Dominican Republic" or Ecuador or Egypt or "El Salvador" or "Equatorial Guinea" or Eritrea or Eswatini or Ethiopia )

ALL=(Fiji or Gabon or Gambia or Georgia or Ghana or Grenada or Guatemala or Guinea or "Guinea Bissau" or Haiti or Honduras or India or Indonesia or Iran or Iraq or Jamaica or Jordan or Kazakhstan or Kenya or Kiribati or Korea or Kosovo or Kyrgyz or Kyrgyzstan or Lao or Lebanon or Lesotho or Liberia or Libya )

ALL=(Pakistan or Palau or Palestine or "Papua New Guinea" or Paraguay or Peru or Philippines or Russia or Rwanda or Samoa or "Sao Tome and Principe" or Senegal or Serbia or "Sierra Leone" or "Solomon Islands" or Somalia or "South Africa" or "South Sudan" or "Sri Lanka" or "St. Lucia" or "St. Vincent and the Grenadines" or Sudan or Suriname or Syria or Tajikistan or Tanzania or Thailand or "Timor-Leste" or Togo or Tonga or Tunisia or Turkiye or Turkey or Turkmenistan or Tuvalu or Uganda or Ukraine or Uzbekistan or Vanuatu or Venezuela or Vietnam or Yemen or Zambia or Zimbabwe)

1. **SCOPUS**

- **Electronic health records:** ( TITLE-ABS-KEY ( ( electronic OR computer* OR digital* ) W/2 ( "health record*" OR "medical record*" OR "nursing record*" OR "nursing document*" OR "document*" OR "patient record*" OR "in-patient record*" OR "inpatient record*" ) ) ) OR ( TITLE-ABS-KEY ( "electronic health record*" ) )
- **Nursing care:** TITLE-ABS-KEY ( "nursing care" OR nurs* )
- **Lower and Middle Income Countries:** ( TITLE-ABS-KEY ( afghanistan OR albania OR algeria OR angola OR argentina OR armenia OR azerbaijan OR bangladesh OR belarus OR belize OR benin OR bhutan OR bolivia OR "Bosnia and Herzegovina" OR botswana OR brazil OR bulgaria OR "Burkina Faso" OR burundi OR "Cabo Verde" OR "Cape Verde" OR cambodia OR cameroon OR "Central African Republic" OR chad OR china OR colombia OR comoros OR congo OR "Costa Rica" OR "Côte d'Ivoire" OR "Ivory Coast" OR cuba OR djibouti OR dominica OR "Dominican Republic" OR ecuador OR egypt OR "El Salvador" OR "Equatorial Guinea" OR eritrea OR eswatini OR ethiopia OR fiji OR gabon OR gambia OR georgia OR ghana OR grenada OR guatemala OR guinea OR "Guinea Bissau" OR haiti OR honduras OR india OR indonesia OR iran OR iraq OR jamaica OR jordan OR kazakhstan OR kenya OR kiribati OR korea OR kosovo OR kyrgyz OR kyrgyzstan OR lao OR lebanon OR lesotho OR liberia OR libya OR madagascar OR malawi OR malaysia OR maldives OR mali OR "Marshall Islands" OR mauritania OR mauritius OR mexico OR micronesia OR moldova OR mongolia OR montenegro OR morocco OR mozambique OR myanmar OR namibia OR nepal OR nicaragua OR niger OR nigeria OR "North Macedonia" OR pakistan OR palau OR palestine OR "Papua New Guinea" OR paraguay OR peru OR philippines OR russia OR rwanda OR samoa OR "Sao Tome and Principe" OR senegal OR serbia OR "Sierra Leone" OR "Solomon Islands" OR somalia OR "South Africa" OR "South Sudan" OR "Sri Lanka" OR "St. Lucia" OR "St. Vincent and the Grenadines" OR sudan OR suriname OR syria OR tajikistan OR tanzania OR thailand OR "Timor-Leste" OR togo OR tonga OR tunisia OR turkiye OR turkey OR turkmenistan OR tuvalu OR uganda OR ukraine OR uzbekistan OR vanuatu OR venezuela OR vietnam OR yemen OR zambia OR zimbabwe ) ) OR ( TITLE-ABS-KEY ( "Low- and middle-income countries" OR "low* and middle income countries" OR "LMICs" OR "LMIC" ) )

1. **GLOBAL INDEX MEDICUS**

- **EHR:** ((electronic OR computer* OR digital*) AND (“health record*” OR “medical record*” OR “nursing record*” OR “nursing document*” OR “document*” OR “patient record*” OR “in-patient record*” OR “inpatient record*”))
- **Nursing care:** (nurs* OR “nursing care”)
- **LMICs:** “Low- and middle-income countries” or “low* and middle income countries” or LMICs or Afghanistan or Albania or Algeria or Angola or Argentina or Armenia or Azerbaijan or Bangladesh or Belarus or Belize or Benin or Bhutan or Bolivia or "Bosnia and Herzegovina" or Botswana or Brazil or Bulgaria or "Burkina Faso" or Burundi or "Cabo Verde" or "Cape Verde" or Cambodia or Cameroon or "Central African Republic" or Chad or China or Colombia or Comoros or Congo or "Costa Rica" or "Côte d'Ivoire" or "Ivory Coast" or Cuba or Djibouti or Dominica or "Dominican Republic" or Ecuador or Egypt or "El Salvador" or "Equatorial Guinea" or Eritrea or Eswatini or Ethiopia or Fiji or Gabon or Gambia or Georgia or Ghana or Grenada or Guatemala or Guinea or "Guinea Bissau" or Haiti or Honduras or India or Indonesia or Iran or Iraq or Jamaica or Jordan or Kazakhstan or Kenya or Kiribati or Korea or Kosovo or Kyrgyz or Kyrgyzstan or Lao or Lebanon or Lesotho or Liberia or Libya or Madagascar or Malawi or Malaysia or Maldives or Mali or "Marshall Islands" or Mauritania or Mauritius or Mexico or Micronesia or Moldova or Mongolia or Montenegro or Morocco or Mozambique or Myanmar or Namibia or Nepal or Nicaragua or Niger or Nigeria or "North Macedonia" or Pakistan or Palau or Palestine or "Papua New Guinea" or Paraguay or Peru or Philippines or Russia or Rwanda or Samoa or "Sao Tome and Principe" or Senegal or Serbia or "Sierra Leone" or "Solomon Islands" or Somalia or "South Africa" or "South Sudan" or "Sri Lanka" or "St. Lucia" or "St. Vincent and the Grenadines" or Sudan or Suriname or Syria or Tajikistan or Tanzania or Thailand or "Timor-Leste" or Togo or Tonga or Tunisia or Turkiye or Turkey or Turkmenistan or Tuvalu or Uganda or Ukraine or Uzbekistan or Vanuatu or Venezuela or Vietnam or Yemen or Zambia or Zimbabwe
