# Supplementary material for: The Impact of Electronic Health Records on Nurses and Nursing Care in Low‐ and Middle‐Income Countries: A Scoping Review
Source: Nurs Open. 2026 Jun 25;13(7):e70649. doi: 10.1002/nop2.70649 (PMC13303348; doi:10.1002/nop2.70649)
Supplement: Supplementary file 2 — Appendix S2: is the MMAT quality appraisal scores of the included studies. [file NOP2-13-e70649-s003.docx]

# Supplementary Material B: The Mixed Methods Appraisal Tool (MMAT version 2018) Score for the Included Studies (n = 41)

| **Authors** | **SQ1** | **SQ2** | **Qual1** | **Qual2** | **Qual3** | **Qual4** | **Qual5** | **QuantC1** | **QuantC2** | **QuantC3** | **QuantC4** | **QuantC5** | **QRCT1** | **QRCT2** | **QRCT3** | **QRCT4** | **QRCT5** |
| --- | --- | --- | --- | --- | --- | --- | --- | --- | --- | --- | --- | --- | --- | --- | --- | --- | --- |
| Abed et al. (2022) | Yes | Yes |  |  |  |  |  | Yes | Yes | Yes | Yes | Yes |  |  |  |  |  |
| Adereti & Olaogun (2019) | Yes | Yes |  |  |  |  |  |  |  |  |  |  |  |  |  |  |  |
| Akhu‐Zaheya et al. (2018) | Yes | Yes |  |  |  |  |  | Yes | Yes | Yes | Yes | Yes |  |  |  |  |  |
| Alfuqaha et al. (2022) | Yes | Yes |  |  |  |  |  | Yes | Yes | Yes | Yes | Yes |  |  |  |  |  |
| Arikan et al. (2022) | Yes | Yes |  |  |  |  |  | Yes | Yes | Yes | Yes | Yes |  |  |  |  |  |
| Attafuah et al. (2022) | Yes | Yes | Yes | No | Yes | Yes | No |  |  |  |  |  |  |  |  |  |  |
| Bei‐lei et al. (2019) | Yes | Yes |  |  |  |  |  | Yes | Yes | Yes | Yes | No |  |  |  |  |  |
| Cheung & Yip (2024) | Yes | Yes |  |  |  |  |  |  |  |  |  |  |  |  |  |  |  |
| Cohen et al. (2016) | Yes | Yes |  |  |  |  |  |  |  |  |  |  |  |  |  |  |  |
| Dolan et al. (2023) | Yes | Yes | Yes | Yes | Yes | Yes | Yes |  |  |  |  |  |  |  |  |  |  |
| Farshi et al. (2015) | Yes | Yes |  |  |  |  |  | Yes | Yes | Yes | No | Yes |  |  |  |  |  |
| Faujdar et al. (2021) | Yes | Yes | Yes | Yes | Yes | Yes | Yes |  |  |  |  |  |  |  |  |  |  |
| Firouzeh et al. (2017) | Yes | Yes |  |  |  |  |  | Yes | Yes | Yes | Yes | Yes |  |  |  |  |  |
| Galani et al. (2021) | Yes | Yes | Yes | Yes | Yes | Yes | Yes |  |  |  |  |  |  |  |  |  |  |
| Gomes et al. (2019) | Yes | Yes | Yes | Yes | Yes | Yes | Yes |  |  |  |  |  |  |  |  |  |  |
| Hariyati et al. (2020) | Yes | Yes |  |  |  |  |  |  |  |  |  |  |  |  |  |  |  |
| Heidarizadeh et al. (2017) | Yes | Yes | Yes | Yes | Yes | Yes | Yes |  |  |  |  |  |  |  |  |  |  |
| Jensen & McKerrow (2022) | Yes | Yes |  |  |  |  |  |  |  |  |  |  |  |  |  |  |  |
| Kahouei, Daimazar, et al. (2015) | Yes | Yes |  |  |  |  |  | Yes | Yes | Yes | Yes | Yes |  |  |  |  |  |
| Kahouei, Zadeh, et al. (2015) | Yes | Yes |  |  |  |  |  | Yes | Yes | Yes | No | Yes |  |  |  |  |  |
| Kamil et al. (2020) | Yes | Yes | Yes | Yes | Yes | Yes | Yes |  |  |  |  |  |  |  |  |  |  |
| Kartika et al. (2021) | Yes | Yes |  |  |  |  |  | Yes | No | Yes | Yes | No |  |  |  |  |  |
| Lei et al. (2023) | Yes | Yes | Yes | Yes | Yes | Yes | Yes |  |  |  |  |  |  |  |  |  |  |
| Mahdizadeh et al. (2022) | Yes | Yes |  |  |  |  |  | Yes | Yes | No | Yes | No |  |  |  |  |  |
| Makeleni & Cilliers (2021) | Yes | Yes | Yes | Yes | Yes | Yes | Yes |  |  |  |  |  |  |  |  |  |  |
| Özer & Şantaş (2020) | Yes | Yes |  |  |  |  |  | Yes | Yes | Yes | No | No |  |  |  |  |  |
| Peivandi et al. (2022) | Yes | Yes |  |  |  |  |  |  |  |  |  |  | Yes | Yes | Yes | No | Yes |
| Peng et al. (2020) | Yes | Yes |  |  |  |  |  |  |  |  |  |  | Yes | Yes | Yes | Yes | Yes |
| Qin et al. (2017) | Yes | Yes | Yes | Yes | Yes | Yes | No |  |  |  |  |  |  |  |  |  |  |
| Selna et al. (2022) | Yes | Yes | Yes | Yes | Yes | Yes | No |  |  |  |  |  |  |  |  |  |  |
| Shafiee et al. (2022) | Yes | Yes |  |  |  |  |  | Yes | Yes | Yes | Yes | Yes |  |  |  |  |  |
| Sinha & Joy (2022) | Yes | Yes |  |  |  |  |  | Yes | Yes | Yes | Yes | Yes |  |  |  |  |  |
| Tierney et al. (2016) | Yes | Yes |  |  |  |  |  | Yes | No | Yes | Yes | Yes |  |  |  |  |  |
| Tilahun & Fritz (2015) | Yes | Yes |  |  |  |  |  | Yes | Yes | Yes | Yes | Yes |  |  |  |  |  |
| Tubaishat (2017) | Yes | Yes |  |  |  |  |  | Yes | Yes | Yes | Yes | Yes |  |  |  |  |  |
| Tubaishat (2019) | Yes | Yes | Yes | Yes | Yes | Yes | Yes |  |  |  |  |  |  |  |  |  |  |
| Uzun & Cerit (2023) | Yes | Yes | Yes | No | Yes | Yes | Yes |  |  |  |  |  |  |  |  |  |  |
| Venkateswaran et al. (2022) | Yes | Yes |  |  |  |  |  |  |  |  |  |  | Yes | Yes | Yes | Yes | Yes |
| Wang et al. (2016) | Yes | Yes |  |  |  |  |  | Yes | Yes | Yes | No | Yes |  |  |  |  |  |
| Yilmaztürk et al. (2023) | Yes | Yes |  |  |  |  |  | No | Yes | Yes | Yes | Yes |  |  |  |  |  |
| Zhai et al. (2022) | Yes | Yes |  |  |  |  |  |  |  |  |  |  |  |  |  |  |  |

***Footnote:*** MMAT = Mixed Methods Appraisal Tool, SQ = Screening question, Qual = Qualitative question, QuantC = Quantitative cross-sectional question, QRCT = Quantitative RCT questions, QNRT = Quantitative non-random trial questions, MM = Mixed method questions.

**Supplementary material B Continued**

| **Authors** | **QNRT1** | **QNRT2** | **QNRT3** | **QNRT4** | **QNRT5** | **MM1** | **MM2** | **MM3** | **MM4** | **MM5** | **Average MMAT score** |
| --- | --- | --- | --- | --- | --- | --- | --- | --- | --- | --- | --- |
| Abed et al. (2022) |  |  |  |  |  |  |  |  |  |  | **5** |
| Adereti & Olaogun (2019) | No | Yes | Yes | Unsure | Yes |  |  |  |  |  | **3** |
| Akhu‐Zaheya et al. (2018) |  |  |  |  |  |  |  |  |  |  | **5** |
| Alfuqaha et al. (2022) |  |  |  |  |  |  |  |  |  |  | **5** |
| Arikan et al. (2022) |  |  |  |  |  |  |  |  |  |  | **5** |
| Attafuah et al. (2022) |  |  |  |  |  |  |  |  |  |  | **3** |
| Bei‐lei et al. (2019) |  |  |  |  |  |  |  |  |  |  | **4** |
| Cheung & Yip (2024) |  |  |  |  |  | Yes | Yes | Yes | Yes | Yes | **5** |
| Cohen et al. (2016) |  |  |  |  |  | Yes | Yes | Yes | No | Yes | **4** |
| Dolan et al. (2023) |  |  |  |  |  |  |  |  |  |  | **5** |
| Farshi et al. (2015) |  |  |  |  |  |  |  |  |  |  | **4** |
| Faujdar et al. (2021) |  |  |  |  |  |  |  |  |  |  | **5** |
| Firouzeh et al. (2017) |  |  |  |  |  |  |  |  |  |  | **5** |
| Galani et al. (2021) |  |  |  |  |  |  |  |  |  |  | **5** |
| Gomes et al. (2019) |  |  |  |  |  |  |  |  |  |  | **5** |
| Hariyati et al. (2020) |  |  |  |  |  | Yes | Yes | Yes | Yes | Yes | **5** |
| Heidarizadeh et al. (2017) |  |  |  |  |  |  |  |  |  |  | **5** |
| Jensen & McKerrow (2022) |  |  |  |  |  | Yes | Yes | Yes | No | Yes | **4** |
| Kahouei, Daimazar, et al. (2015) |  |  |  |  |  |  |  |  |  |  | **5** |
| Kahouei, Zadeh, et al. (2015) |  |  |  |  |  |  |  |  |  |  | **2** |
| Kamil et al. (2020) |  |  |  |  |  |  |  |  |  |  | **5** |
| Kartika et al. (2021) |  |  |  |  |  |  |  |  |  |  | **3** |
| Lei et al. (2023) |  |  |  |  |  |  |  |  |  |  | **5** |
| Mahdizadeh et al. (2022) |  |  |  |  |  |  |  |  |  |  | **3** |
| Makeleni & Cilliers (2021) |  |  |  |  |  |  |  |  |  |  | **5** |
| Özer & Şantaş (2020) |  |  |  |  |  |  |  |  |  |  | **3** |
| Peivandi et al. (2022) |  |  |  |  |  |  |  |  |  |  | **4** |
| Peng et al. (2020) |  |  |  |  |  |  |  |  |  |  | **5** |
| Qin et al. (2017) |  |  |  |  |  |  |  |  |  |  | **4** |
| Selna et al. (2022) |  |  |  |  |  |  |  |  |  |  | **4** |
| Shafiee et al. (2022) |  |  |  |  |  |  |  |  |  |  | **5** |
| Sinha & Joy (2022) |  |  |  |  |  |  |  |  |  |  | **5** |
| Tierney et al. (2016) |  |  |  |  |  |  |  |  |  |  | **4** |
| Tilahun & Fritz (2015) |  |  |  |  |  |  |  |  |  |  | **5** |
| Tubaishat (2017) |  |  |  |  |  |  |  |  |  |  | **5** |
| Tubaishat (2019) |  |  |  |  |  |  |  |  |  |  | **5** |
| Uzun & Cerit (2023) |  |  |  |  |  |  |  |  |  |  | **4** |
| Venkateswaran et al. (2022) |  |  |  |  |  |  |  |  |  |  | **5** |
| Wang et al. (2016) |  |  |  |  |  |  |  |  |  |  | **4** |
| Yilmaztürk et al. (2023) |  |  |  |  |  |  |  |  |  |  | **4** |
| Zhai et al. (2022) |  |  |  |  |  | Yes | Yes | Yes | Yes | Yes | **5** |

***Footnote:*** MMAT = Mixed Methods Appraisal Tool, SQ = Screening question, Qual = Qualitative question, QuantC = Quantitative cross-sectional question, QRCT = Quantitative RCT questions, QNRT = Quantitative non-random trial questions, MM = Mixed method questions.

**MMAT appraisal Key. NB: Answers to these questions were either “Yes”, “No” or “Unsure**

*Q1 = Are there clear research questions?*

*SQ2 = Do the collected data address the research questions?*

*Qual1 = Is the qualitative approach appropriate to answer the research question?*

*Qual2 = Are the qualitative data collection methods adequate to address the research question?*

*Qual3 = Are the findings adequately derived from the data?*

*Qual4 = Is the interpretation of results sufficiently substantiated by data?*

*Qual5 = Is there coherence between qualitative data sources, collection, analysis and interpretation?*

*QuantC1 = Is the sampling strategy relevant to address the research question?*

*QuantC2 = Is the sample representative of the target population?*

*QuantC3 = Are the measurements appropriate?*

*QuantC4 = Is the risk of nonresponse bias low?*

*QuantC5 = Is the statistical analysis appropriate to answer the research question?*

*QRCT1 = Is randomization appropriately performed?*

*QRCT2 = Are the groups comparable at baseline?*

*QRCT3 = Are there complete outcome data?*

*QRCT4 = Are outcome assessors blinded to the intervention provided?*

*QRCT5 = Did the participants adhere to the assigned intervention?*

*QNRT1 = Is randomization appropriately performed?*

*QNRT2 = Are the groups comparable at baseline?*

*QNRT3 = Are there complete outcome data?*

*QNRT4 = Are outcome assessors blinded to the intervention provided?*

*QNRT5 = Did the participants adhere to the assigned intervention?*

*MM1 = Is there an adequate rationale for using a mixed methods design to address the research question?MM2 =*

*MM2 = Are the different components of the study effectively integrated to answer the research question?*

*MM3 = Are the outputs of the integration of qualitative and quantitative components adequately interpreted?*

*MM4 = Are divergences and inconsistencies between quantitative and qualitative results adequately addressed?*

*MM5 = Do the different components of the study adhere to the quality criteria of each tradition of the methods involved?*

***Average MMAT Score*** *= This is the total average score of the paper type.*

***Interpretation:***

*Average MMAT score interpretation key:*

- ***4 or 5 = High methodological quality***
- ***3 = Medium methodological quality***
- ***0 or 2 = Poor methodological quality***
