# Supplementary material for: The Impact of Electronic Health Records on Nurses and Nursing Care in Low‐ and Middle‐Income Countries: A Scoping Review
Source: Nurs Open. 2026 Jun 25;13(7):e70649. doi: 10.1002/nop2.70649 (PMC13303348; doi:10.1002/nop2.70649)
Supplement: Supplementary file 4 — Appendix S4: contains the theories and theoretical frameworks used by the included studies. [file NOP2-13-e70649-s002.docx]

# Supplementary material D: Theories and Models Used in the Included Studies (n = 41)

| **Authors & year** | **Title** | **Country** | **Name of theory or model used** |
| --- | --- | --- | --- |
| Abed et al. (2022) | Jordanian nurses’ attitudes toward using electronic health records during COVID-19 pandemic Using EHRs During COVID-19 in Jordan | Jordan | N/A |
| Adereti & Olaogun (2019) | Use of Electronic and Paper-based Standardized Nursing Care Plans to Improve Nurses' Documentation Quality in a Nigerian Teaching Hospital | Nigeria | N/A |
| Akhu‐Zaheya et al. (2018) | Quality of nursing documentation: Paper-based health records versus electronic-based health records | Jordan | The VIPS model, VIPS model—a model designed to structure nursing documentation. |
| Alfuqaha et al. (2022) | Technology acceptance model among nurses and other healthcare providers during the 2019 Coronavirus pandemic: a comparative cross-sectional study | Jordan | Technology Acceptance Model (TAM) by Davis (1989) |
| Arikan et al. (2022) | Barriers to Adoption of Electronic Health Record Systems from the Perspective of Nurses | Turkey | N/A |
| Attafuah et al. (2022) | Satisfied or not satisfied? Electronic health records system implementation in Ghana: Health leaders’ perspective | Ghana | N/A |
| Bei‐lei et al. (2019) | Current status and nurses' perceptions of the electronic tabular nursing records in Henan, China | China | N/A |
| Cheung & Yip (2024) | Documentation Completeness and Nurses' Perceptions of a Novel Electronic App for Medical Resuscitation in the Emergency Room: Mixed Methods Approach | China | N/A |
| Cohen et al. (2016) | An importance-performance analysis of hospital information system attributes: A nurses' perspective | South Africa | The DeLone and McLean Information Systems Success Model (DeLone & McLean, 1992; 2003) |
| Dolan et al. (2023) | Integration of a Digital Health Intervention into Immunization Clinic Workflows in Kenya: Qualitative, Realist Evaluation of Technology Usability | Kenya | 1. The Fit Between Individual, Task, and Technology framework (Ammenerth et al., 2006). 2. The Ergonomics Balance Theory (Smith & Sainfort, 1989). |
| Farshi et al. (2015) | Comparison of Manual and Electronic Methods of Nursing Record: A Nurse's Perspective | Iran | N/A |
| Faujdar et al. (2021) | Stakeholders' Perceptions of the Implementation of a Patient-Centric Digital Health Application for Primary Healthcare in India | India | 1. Theory of Interpersonal Behaviour (TIB) by Gagnon et al. (2008)  2. The COM-B model by Michie et al. (2011). |
| Firouzeh et al. (2017) | Evaluation of vocal-electronic nursing documentation: A comparison study in Iran | Iran | N/A |
| Galani et al. (2021) | Improving continuity of HIV/AIDS care through electronic health records in resource-limited settings: A Botswana perspective | Botswana | Constructivist grounded theory by Tie et al. (2019) |
| Gomes et al. (2019) | Electronic Citizen Record: An Instrument for Nursing Care | Brazil | N/A |
| Hariyati et al. (2020) | Usability and satisfaction of using electronic nursing documentation, lesson-learned from new system implementation at a hospital in Indonesia | Indonesia | Lewin's Force Field Analysis model |
| Heidarizadeh et al. (2017) | Nurses' Perception of Challenges in the Use of an Electronic Nursing Documentation System | Iran | Technology Acceptance Model 2 (TAM2) by Venkatesh and Davis (2000) |
| Jensen & McKerrow (2022) | Acceptability and uptake of an electronic decision-making tool to support the implementation of IMCI in primary healthcare facilities in KwaZulu-Natal, South Africa | South Africa | N/A |
| Kahouei, Daimazar, et al. (2015) | The evaluation of the compatibility of electronic patient record (EPR) system with nurses' management needs in a developing country | Iran | N/A |
| Kahouei, Zadeh, et al. (2015) | Path analysis model of nursing staffs' experiences of EPR benefits | Iran | N/A |
| Kamil et al. (2020) | Exploring Health Professionals' Perceptions on Health-ID, an Electronic Integrated Patient Progress Documentation System: A Qualitative Study in Indonesia | Indonesia | N/A |
| Kartika et al. (2021) | Nurses and physicians' perceptions on the Electronic Health Record implementation | Indonesia | Socio-technical model by Sittig and Singh (2010) |
| Lei et al. (2023) | Adopting an electronic medication administration system in long-term care facilities: a key stakeholder interview study in Macao | China | N/A |
| Mahdizadeh et al. (2022) | Challenges and Opportunities of Using the National Electronic Health Record from the Perspective of Iranian Nursing Staff | Iran | N/A |
| Makeleni & Cilliers (2021) | Critical success factors to improve data quality of electronic medical records in public healthcare institutions | South Africa | The Data Quality Framework (DQF) by Smartbridge (2024) |
| Özer & Şantaş (2020) | Effects of electronic medical records on patient safety culture: The perspective of nurses | Turkey | N/A |
| Peivandi et al. (2022) | Evaluation and comparison of errors on nursing notes created by online and offline speech recognition technology and handwritten: an interventional study | Iran | N/A |
| Peng et al. (2020) | Early warning of nursing risk based on patient electronic medical record information | China | N/A |
| Qin et al. (2017) | The effect of nursing participation in the design of a critical care information system: a case study in a Chinese hospital | China | N/A |
| Selna et al. (2022) | Challenges to using electronic health records to enhance patient safety, in a Small Island Developing State (SIDS) context | Maldives | N/A |
| Shafiee et al. (2022) | Development and evaluation of an electronic nursing documentation system | Iran | N/A |
| Sinha & Joy (2022) | Nurses’ knowledge of and attitude to nursing information systems | India | N/A |
| Tierney et al. (2016) | Assessing the impact of a primary care electronic medical record system in three Kenyan rural health centers | Kenya | N/A |
| Tilahun & Fritz (2015) | Comprehensive Evaluation of Electronic Medical Record System Use and User Satisfaction at Five Low-Resource Setting Hospitals in Ethiopia | Ethiopia | The DeLone and MacLean information system success evaluation model (DeLone & MacLean, 2003) |
| Tubaishat (2017) | Evaluation of Electronic Health Record Implementation in Hospitals | Jordan | DeLone and McLean Information Systems Success model by DeLone and McLean (1992) |
| Tubaishat (2019) | The effect of electronic health records on patient safety: A qualitative exploratory study | Jordan | N/A |
| Uzun & Cerit (2023) | Effect of Digitalization on Nursing Practices Using the Lean Approach | Turkey | The Lean approach, which originated from the Toyota Production System. |
| Venkateswaran et al. (2022) | eRegTime - Time Spent on Health Information Management in Primary Health Care Clinics Using a Digital Health Registry Versus Paper-Based Documentation: Cluster-Randomized Controlled Trial | Palestine | N/A |
| Wang et al. (2016) | Making Patient Risk Visible: Implementation of a Nursing Document Information System to Improve Patient Safety | China | N/A |
| Yilmaztürk et al. (2023) | The effect of digitalization of nursing forms in ICUs on time and cost | Turkey | The Electronic Medical Record Adoption Model (EMRAM) developed by the Healthcare Information and Management Systems Society (HIMSS) |
| Zhai et al. (2022) | Transition to a new nursing information system embedded with clinical decision support: a mixed method study using the HOT-fit framework | China | 1. DeLone and McLean Information Systems Success model by DeLone and McLean (2003)  2. The Human, Organization and Technology Fit (HOT-fit) model (Yusof et al., 2008) |

*Footnote:* N/A = Not applicable
